# Cancer‐associated fibroblast‐derived colony‐stimulating factor 2 confers acquired osimertinib resistance in lung adenocarcinoma via promoting ribosome biosynthesis

**DOI:** 10.1002/mco2.653

**Published:** 2024-07-20

**Authors:** Yutang Huang, Xiaoqing Wang, Chunjie Wen, Jingchan Wang, Honghao Zhou, Lanxiang Wu

**Affiliations:** ^1^ Institute of Life Sciences Chongqing Medical University Chongqing China; ^2^ School of Stomatology Chongqing Medical University Chongqing China; ^3^ Pharmacogenetics Research Institute Institute of Clinical Pharmacology Central South University Changsha China

**Keywords:** cancer‐associated fibroblasts, colony‐stimulating factor 2, lung adenocarcinoma, Osimertinib resistance, ribosome biosynthesis

## Abstract

Acquired resistance is a major obstacle to the therapeutic efficacy of osimertinib in lung adenocarcinoma (LUAD), but the underlying mechanisms are still not fully understood. Cancer‐associated fibroblasts (CAFs) are the most abundant stromal cell type in LUAD tumor‐microenvironment (TME) and have emerged as a key player in chemoresistance. However, the function of CAFs in osimertinib resistance is still unclear. Here, we showed that CAFs derived from osimertinib‐resistant LUAD tissues (CAF^OR^) produced much more colony‐stimulating factor 2 (CSF2) than those isolated from osimertinib‐sensitive tissues. CAF^OR^‐derived CSF2 activated the Janus kinase 2 (JAK2)/Signal transducer and activator of transcription 3 (STAT3) signaling pathway and upregulated lnc‐CSRNP3 in LUAD cells. Lnc‐CSRNP3 then promoted the expression of nearby gene *CSRNP3* by recruiting chromodomain helicase DNA binding protein 9 (CHD9) and inhibited the phosphatase activity of the serine/threonine protein phosphatase 1 catalytic subunit α (PP1α), thereby induced osimertinib resistance by enhancing ribosome biogenesis. Collectively, our study reveals a critical role for CAFs in the development of osimertinib resistance and identifies the CSF2 pathway as an attractive target for monitoring osimertinib efficacy and overcoming osimertinib resistance in LUAD.

## Graphical abstract

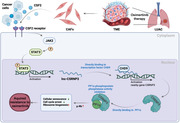

## 1 INTRODUCTION

Lung cancer is the leading cause of cancer‐related death globally, with lung adenocarcinoma (LUAD) accounting for around 50% of all diagnosed cases.^1^, ^2^ It is well established that LUAD patients harboring activating mutations in the epidermal growth factor receptor (EGFR), one of the major oncogenic drivers of LUAD, exhibit a better prognosis when treated with EGFR tyrosine kinase inhibitors (EGFR‐TKIs) compared with those treated with standard platinum‐based chemotherapy.^3^, ^4^ However, although the first generation of EGFR‐TKIs, such as gefitinib and erlotinib, had led to notable clinical efficacy in LUAD patients, acquired resistance emerged after 10−14 months of treatment, which is partially attributed to the secondary EGFR^T790M^ mutation.^5^, ^6^ Therefore, the third‐generation EGFR‐TKI, osimertinib, was developed specifically to target the EGFR^T790M^ mutation, but newly developed acquired resistance is still inevitable, hindering its clinical application.^7^ As a result, the potential biomarkers and therapeutic targets to monitor and overcome osimertinib resistance are urgently required.

Recently, the roles of the tumor microenvironment (TME) in tumorigenesis and chemoresistance have attracted increasing attention.^8^ Cancer‐associated fibroblasts (CAFs), the primary component of the tumor stroma, undergo metamorphosis and demonstrate heterogeneity during tumor progression, have been shown to contribute to almost every aspect of tumor progression, providing an attractive therapeutic target.^9^ To date, different CAF populations have been identified in LUAD,^10^, ^11^ which are indicated to be involved in the resistance to several anti‐tumor drugs.^12^, ^13^, ^14^ However, only a few studies have investigated the association between CAFs and osimertinib resistance in LUAD. Thus, the precise roles and underlying mechanisms of CAFs in the development of acquired osimertinib resistance in LUAD still remain to be elucidated.

Colony‐stimulating factor 2 (CSF2), also known as granulocyte macrophage‐colony stimulating factor, is a cytokine that encourages stem cells to generate granulocytes and monocytes.^15^, ^16^ As previously reported, CSF2 directly binds to the stimulating factor 2 receptor (CSF2R) and activates four main pathways in cancer cells, including Janus kinase 2 (JAK2)/Akt, JAK2/Signal transducer and activator of transcription (STAT), Mitogen‐activated protein kinase, and nuclear factor (NF)‐кB pathways, and plays an important role in tumorigenesis and chemoresistance.^17^, ^18^ For example, serum CSF2 level has been demonstrated to be increased in patients with lung cancer, and CSF2‐dependent activation of the JAK2/signal transducer and activator of transcription 3 (STAT3) pathway is important in tumor angiogenesis and vascularization.^19^, ^20^ In addition, CSF2 also plays a role in resistance to oxaliplatin and 5‐fluorouracil in gallbladder and colorectal cancers.^21^, ^22^ However, the exact role of CSF2 in the development of acquired osimertinib resistance in LUAD is still unclear.

Here, we aimed to obtain a better understanding of CAFs‐induced osimertinib resistance in LUAD to identify novel therapeutic targets for overcoming this phenomenon. Furthermore, we aimed to identify a promising circulating biomarker for monitoring the occurrence and development of osimertinib resistance during treatment in LUAD patients.

## 2 RESULTS

### 2.1 Single nucleus cell profiling of osimertinib‐sensitive and osimertinib‐resistant LUAD tissues

**FIGURE 1:**
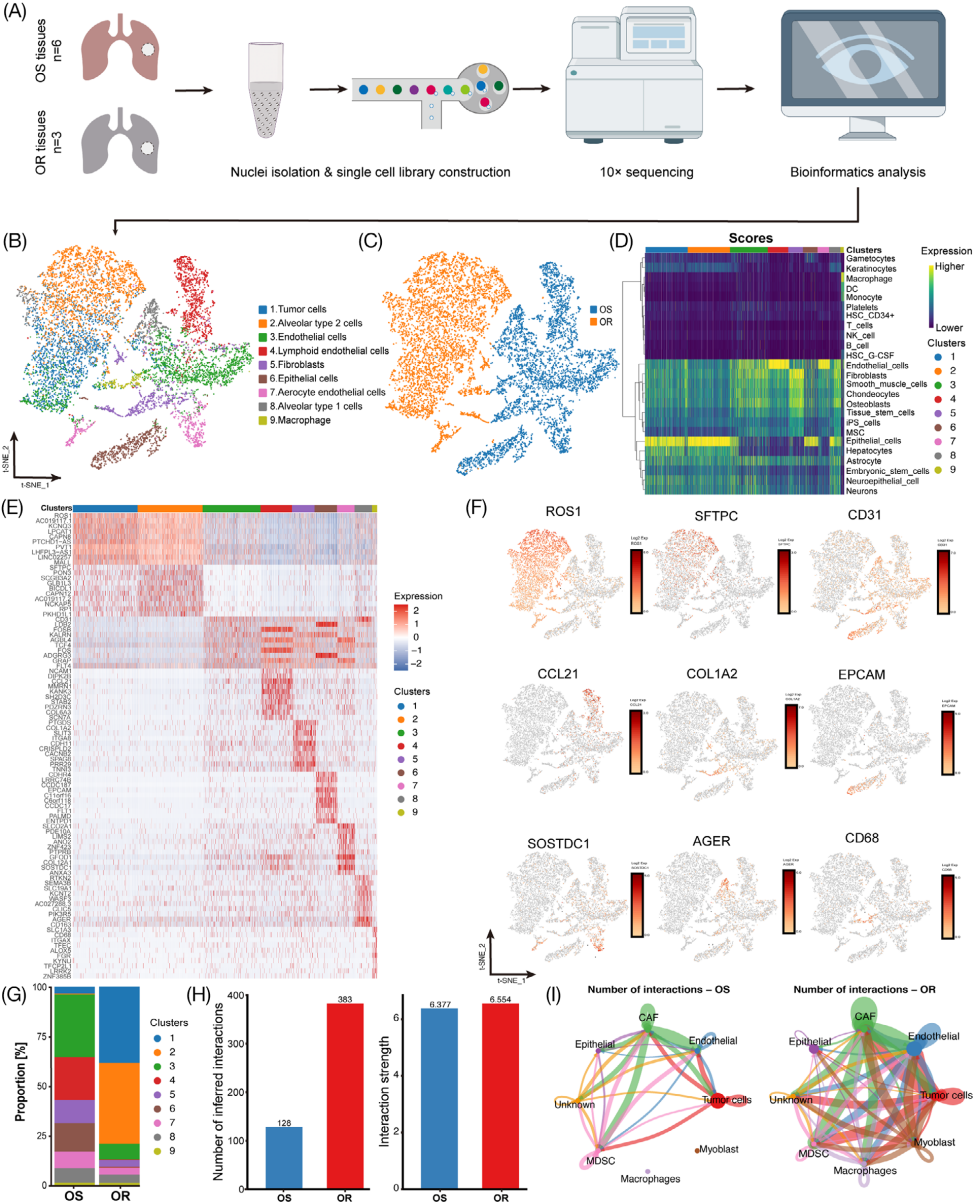
Comprehensive cellular overview and crosstalk in osimertinib‐sensitive and osimertinib‐resistant LUCA tumor microenvironment. (A) Overview of snRNA‐seq using osimertinib‐sensitive (OS) and osimertinib‐resistant (OR) frozen tumor tissue samples. t‐Distributed stochastic neighbor embedding (t‐SNE) plots displaying 12,061 cell profiles with each cell color‐coded for (B) associated cell type and (C) its sample origin. (D) Correlation analysis heatmap for cell type identification. (E) The heatmap shows differentially expressed marker genes (rows) in nine clusters. Red and blue: high and low expression, respectively. (F) t‐SNE plot for marker gene expression for *ROS1* (cluster 1, C1), *SFTPC* (C2), *CD31* (C3), *CCL21* (C4), *COL1A2* (C5), *EPCAM* (C6), *SOSTDC1* (C7), *AGER* (C8) and *CD68* (C9). (G) Cell population proportion between OS and OR samples. (H) Cell chat analysis between OS and OR samples. Left: number of inferred interactions; right: interaction strength. (I) Interaction number network plot in OS and OR samples.

The single‐cell atlas of the LUAD tissues was characterized using nine surgical frozen samples comprising six osimertinib‐sensitive LUAD samples and three osimertinib‐resistant LUAD samples. Each sample was processed to isolate a single nucleus without prior selection of cell types, and then single‐nucleus RNA sequencing (snRNA‐seq) was performed using a 10× Genomics Chromium platform to generate RNA‐seq data. After quality filtering, 12,061 high‐quality cells with a median of 1006–1367 genes per cell were analyzed (Figure 1A, Table S1). These cells were further identified to be nine separate cell types, including tumor cells (22%), alveolar type 2 cells (21%), endothelial cells (19%), lymphoid endothelial cells (10%), fibroblasts (7%), epithelial cells (7%), aerocyte endothelial cells (6%), alveolar type 1 cells (6%), and macrophage (2%) (Figure 1B). The *t*‐SNE plot also showed distinct clustering according to the tumor origin (Figure 1C), and the heatmap depicted the differentially expressed marker genes in nine clusters (Figure 1D).

The cell type composition and their proportions showed a high degree of variation between osimertinib‐sensitive and osimertinib‐resistant LUAD tissues. Immune cells mainly included macrophages (CD68^+^), nonimmune cells mainly included tumor cells (ROS1^+^), fibroblasts (COL1A2^+^), alveolar type 2 epithelial cells (SFTPC^+^), alveolar type 1 epithelial cells (AGER^+^), epithelial cells (EPCAM^+^), endothelial cells (CD31^+^), lymphoid endothelial cells (CCL21^+^), and aerocyte endothelial cells (SOSTDC1^+^) (Figure 1E–G). With Cell chat analysis, we established a global cell‐to‐cell communication network among most of the cell types, including CAFs, endothelial cells, epithelial cells, macrophages, tumor cells, etc (Figure S1A). The number of cell‐to‐cell interactions (ligand‐receptor pairs) was significantly higher in the OR group compared with the OS group (383 and 128, respectively), whereas the strength of these interactions was almost similar (6.554 and 6.377, respectively) (Figure 1H). Moreover, the number of interactions between different cell populations, especially the CAF–tumor cell interactions, was significantly higher in osimertinib‐resistant tissues compared with sensitive ones, including the CSF2‐CSF2RA pair, CCL20‐CCR6 pair, etc. (Figure 1I; Figure S1B, C). Collectively, these data suggest that CAF–tumor cell interactions might participate in the development of osimertinib resistance.

### 2.2 CAFs were correlated with osimertinib resistance in LUAD

**FIGURE 2:**
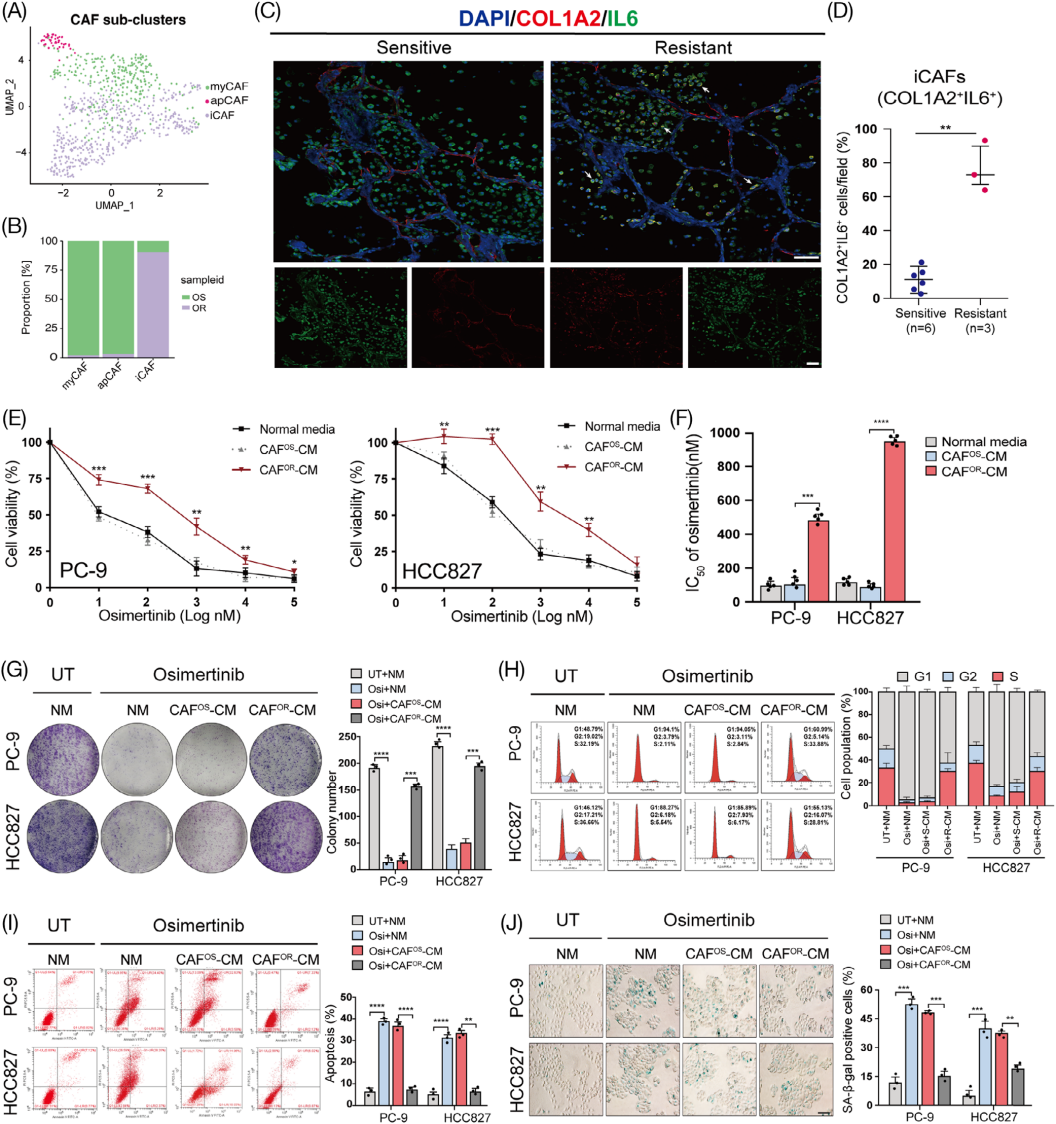
iCAFs were abundant and correlated with osimertinib resistance in LUCA. (A) Re‐clustering of 775 fibroblast cells color‐coded by clusters. (B) Subcluster cell population proportion between OS and OR samples. (C) Representative immunofluorescence images of LUAD tissues before and after osimertinib resistance. Arrows indicate iCAFs: PDPN^+^ IL6^+^. Scale bar = 100 µm. (D) Quantification of the presence of iCAFs (*n *= 6 tumor tissue samples from OS patients or 3 from OR patients). PC‐9 and HCC827 cells were cultured under CAF^OS^2‐CM or CAF^OR^2‐CM for 3 days and then subjected to the indicated experiments. (E–F) Cells were treated with the indicated concentration of osimertinib for 48 h. Cell viability was measured by CCK‐8 and the IC_50_ value was calculated. (G) Colony formation assay, (H) flow cytometry cell cycle analyses, (I) flow cytometry apoptosis analyses, and (J) SA‐β‐Gal staining assays were performed to evaluate the osimertinib sensitivity of LUAD cells in each group. The results are presented as the mean ± SD of three technical replicates, and each dot represents a technical replicate of the assay. *p < 0.05; **p < 0.01; ***p < 0.001; ****p < 0.0001, ns = no significance.

Previous research has identified three types of CAFs, including inflammatory CAFs (iCAFs), myofibroblastic CAFs (myCAFs), and antigen‐presenting CAFs (apCAFs). iCAFs are primarily distinguished by the release of inflammatory substances such as interleukin‐6 (IL‐6), leukemia inhibitory factor (LIF), and interleukin 1 (IL‐1). MyCAFs are characterized by high actin alpha 2 (α‐SMA), transforming growth factor‐β (TGF‐β), and extracellular matrix expression, while apCAFs commonly express major histocompatibility complex class II (MHC class II) and class II transactivator (CD74) molecules.^23^, ^24^ In this study, we performed a re‐clustering of 812 fibroblasts and assigned each of these subclusters based on known markers (Figure S2). Consistent with previous research, we also identified myCAFs (COL1A2^+^αSMA^+^), apCAFs (COL1A2^+^MHCII^+^), and iCAFs (COL1A2^+^IL6^+^) (Figure 2A). Interestingly, iCAFs were significantly enriched in the osimertinib‐resistant tissues compared with sensitive ones (Figure 2B). Moreover, immunofluorescence analysis also confirmed that the proportion of iCAFs was significantly higher in resistant tissues, indicating that CAFs assume an iCAF phenotype after osimertinib resistance. (Figure 2C, D).

To determine whether CAFs contribute to osimertinib resistance, they were isolated from osimertinib‐sensitive (CAF^OS^) or osimertinib‐resistant (CAF^OR^) LUAD tissues and cultured in vitro. Then, LUAD cell lines (PC‐9 and HCC827) were treated with conditioned medium from CAF^OS^ (CAF^OS^‐CM) and CAF^OR^ (CAF^OR^‐CM). Interestingly, compared with CAF^OS^‐CM, CAF^OR^‐CM treatment significantly increased the viability and colony‐formation capacity of LUAD cells after osimertinib exposure (Figure 2E–G). Furthermore, CAF^OR^‐CM dramatically abolished the osimertinib‐induced cell cycle arrest, apoptosis, and senescence in LUAD cells (Figure 2H–J). Taken together, our data suggest that CAF^OR^‐CM confers osimertinib resistance in LUAD cells.

### 2.3 CSF2 was required for CAF^OR^‐induced osimertinib resistance in LUAD cells

**FIGURE 3:**
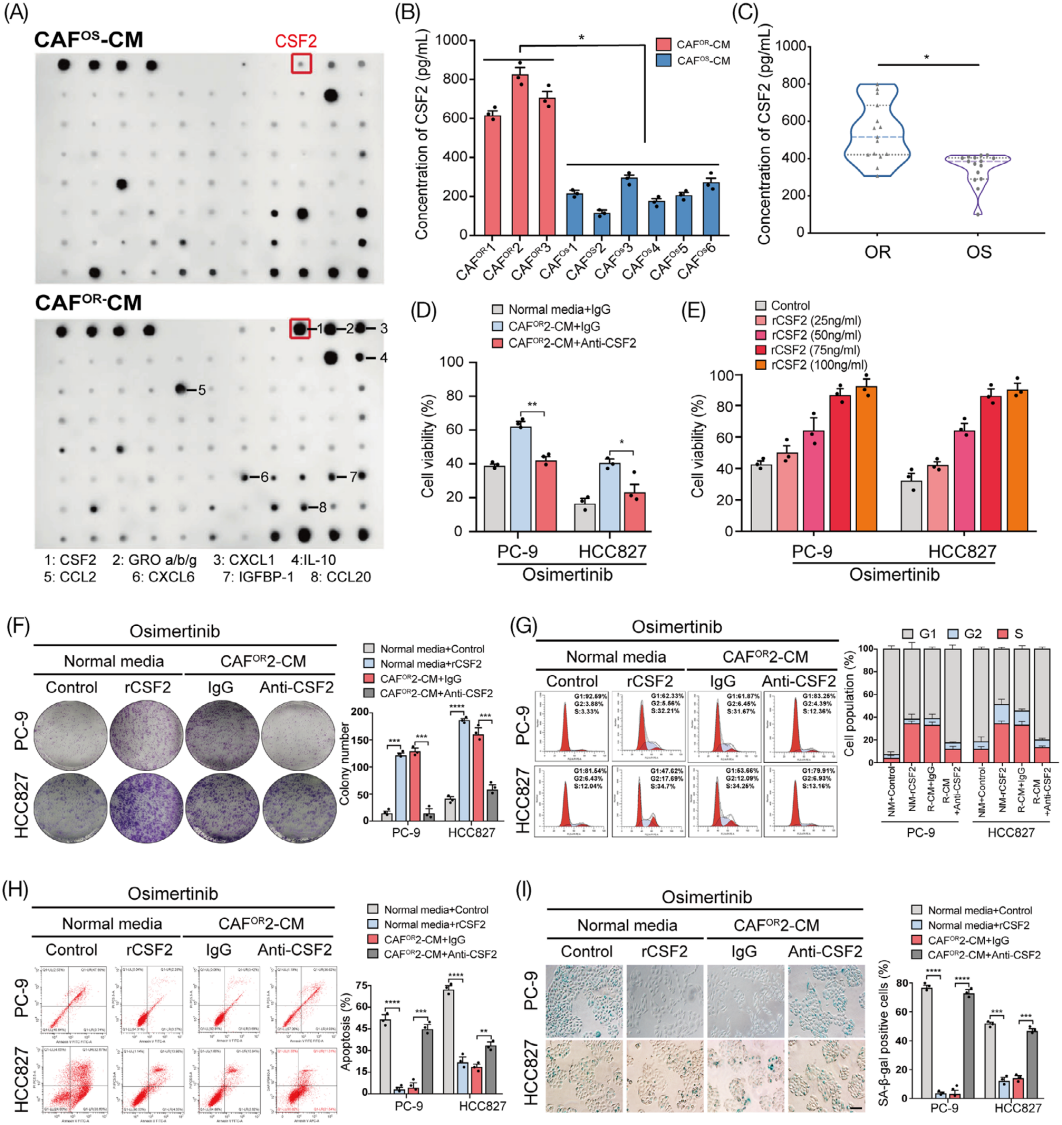
CSF2 was essential for CAF^OR^‐induced osimertinib resistance in tumor cells. (A) Cytokine antibody array of CAF^OS^2‐CM or CAF^OR^2‐CM. (B) ELISA analysis of the CSF2 levels in CAF^OS^‐CM and CAF^OR^‐CM of nine different osimertinib‐sensitive and osimertinib‐resistant CAF cell lines. (C) The expression of CSF2 in the plasma of osimertinib‐sensitive and osimertinib‐resistant LUAD patients was examined by ELISA. (D) CAF^OR^2‐CM with CSF2 neutralizing antibodies was used to culture PC‐9 and HCC827 cells for 3 days. After 48 h of 500 nM osimertinib exposure, cell viability was measured by CCK8. (E) PC‐9 and HCC827 cells were cultured with different concentrations of human recombinant CSF2 (rCSF2) for 3 days and subsequently treated with 500 nM osimertinib for 48 h. Cell viability was measured by CCK8. PC‐9 and HCC827 cells were cultured with CSF2 (100 ng/mL) or CAF^OR^2‐CM with an anti‐CSF2 neutralizing antibody (250 ng/mL) for 3 days. Colony formation assay (F), flow cytometry cell cycle analysis (G), flow cytometry apoptosis analysis (H), and SA‐β‐Gal staining assays (I) were performed to evaluate the osimertinib sensitivity of LUAD cells in each group. The results are presented as the mean ± SD of three technical replicates, and each dot represents a technical replicate of the assay. *p < 0.05; **p < 0.01; ***p < 0.001; ****p < 0.0001, ns = no significance.

To determine which “ligand‐receptor pair” mediates the interaction between CAF^OR^ and LUAD cells, we conducted the human cytokine array to explore the cytokine profiles of CAF^OR^‐CM and CAF^OS^‐CM and identified higher levels of CSF2 in CAF^OR^‐CM (Figure 3A; Figure S3A, B). The enzyme‐linked immunosorbent assay was further performed to measure the concentration of CSF2 in the culture medium of CAF^OR^ and CAF^OS^. As expected, CAF^OR^ secreted much more CSF2 than CAF^OS^, but primary tumor cells isolated from osimertinib‐sensitive and osimertinib‐resistant LUAD tissues showed no difference in CSF2 expression and secretion (Figure 3B; Figure S3C, D). To assess the clinical significance of CSF2, we detected the level of CSF2 in the plasma samples of osimertinib‐sensitive and osimertinib‐resistant LUAD patients and found that the CSF2 level was significantly correlated with the development of osimertinib resistance, showing about 10.32‐fold higher in resistant patients than that in sensitive ones (Figure 3C; Figure S3E, F; Table S2). These data together show that CSF2 might be a potential monitoring biomarker for the acquired resistance to osimertinib in LUAD.

Then, by adding a neutralizing antibody against CSF2, we found that blocking CSF2 successfully abrogated CAF^OR^‐CM‐induced increase in cell viability following osimertinib exposure in a dose‐dependent manner (Figure 3D, E). In addition, the anti‐CSF2 antibody also significantly rescued the influences of CAF^OR^‐CM on colony formation, cell cycle, apoptosis, and senescence in LUAD cells under osimertinib treatment (Figure 3F–I). Collectively, these results indicate that CSF2 plays a critical role in CAF^OR^‐induced osimertinib resistance in LUAD cells.

### 2.4 CAF^OR^‐derived CSF2 facilitated osimertinib resistance by promoting the expression of lnc‐CSRNP3 in LUAD cells

**FIGURE 4:**
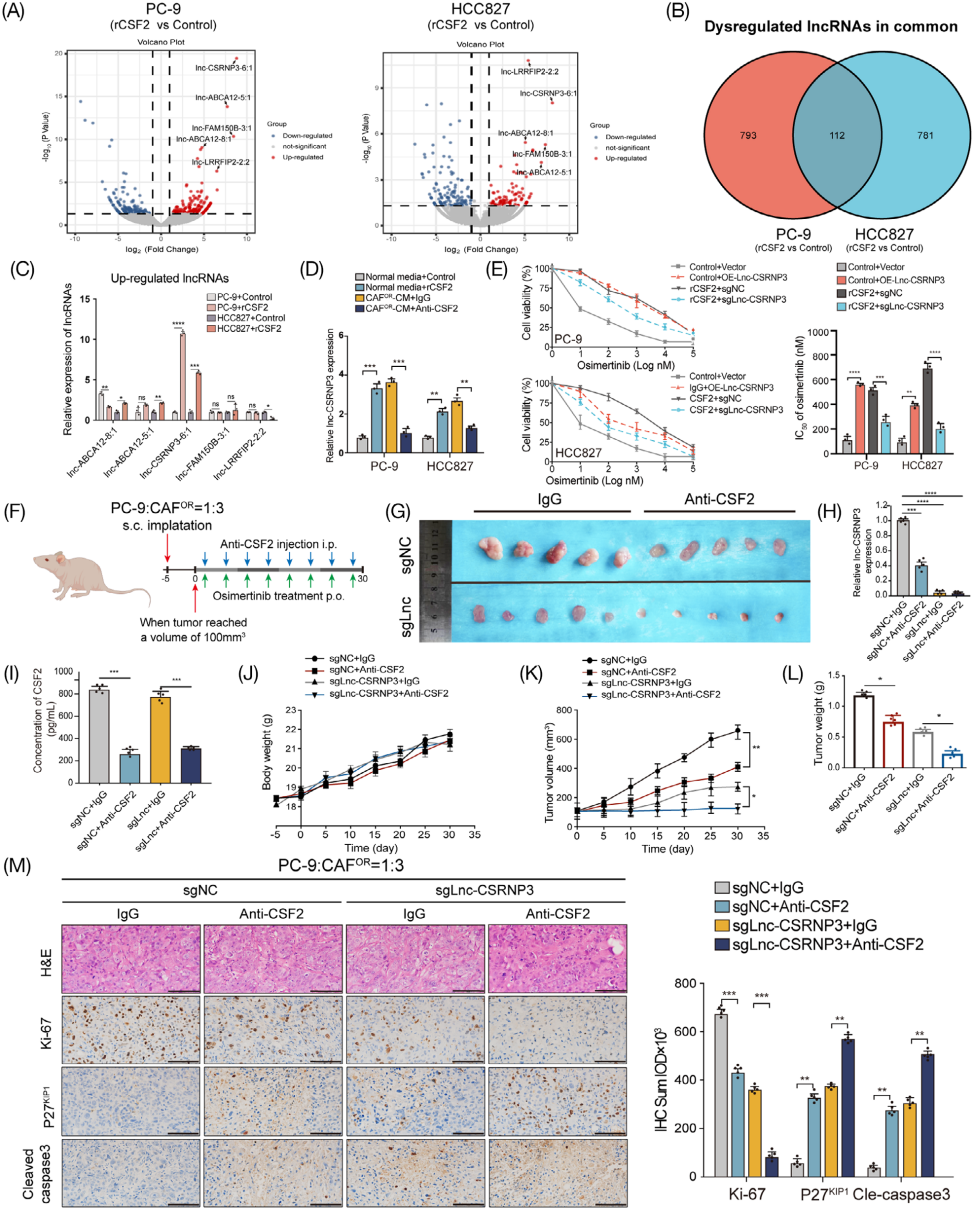
CSF2 facilitated osimertinib resistance by activating the expression of the lncRNA lnc‐CSRNP3 in vitro and in vivo. (A) Volcano plot showing the differentially expressed lncRNAs (|Log 2 FC | > 1, *p*‐value < 0.05) in the RNA sequencing data of PC‐9 and HCC827 cells treated with rCSF2 (CSF2‐Fc fusion protein) or control (hIgG1 Fc fragments). (B) Venn diagram showed the number of differentially expressed lncRNAs overlapped between two comparisons. (C) The expression of the TOP5 common upregulated lncRNAs was validated using qRT‐PCR. Each dot represents a technical replicate of the assay (D) Lnc‐CSRNP3 expression levels in PC‐9 and HCC827 cells cultured under rCSF2 (100 ng/mL) or CAF^OR^2‐CM with an anti‐CSF2 neutralizing antibody (250 ng/mL) for 3 days. Each dot represents a technical replicate of the assay (E) Lnc‐CSRNP3 was overexpressed in LUAD cells cultured with control fragments or knockout in LUAD cells cultured with rCSF2, and osimertinib was given. CCK‐8 assay was performed to evaluate the osimertinib sensitivity of LUAD cells in each group. Each dot represents a technical replicate of the assay. (F) Once the tumors reached 100^3^ mm in volume, the xenograft subcutaneous model received an anti‐CSF2 neutralizing antibody (20 mg/kg, i.p.) and osimertinib (10 mg/kg, p.o.) twice a week. (G) Representative image of tumors from different groups in xenograft subcutaneous model (*n *= 5 mice). (H) The relative expression of lnc‐CSRNP3 in the subcutaneous model of xenografts in various groups (*n *= 5 mice). (I) The concentration of CSF2 in the subcutaneous model of xenografts in various groups (*n *= 5 mice). (J) Average body weight curves of xenograft subcutaneous model in different groups (*n *= 5 mice). (K, L) Tumor growth of subcutaneous model in different groups (*n *= 5 mice). (M) H&E staining in xenograft tumors. Scale bar = 100 µm; (*n *= 5 mice). Representative immunohistochemical staining of Ki‐67, P27^KIP1^, cle‐caspase3 in the subcutaneous model (scale bar = 100 µm; *n *= 5 mice). *p < 0.05; **p < 0.01; ***p < 0.001; ****P < 0.0001, ns = no significance.

To molecularly dissect how CAF^OR^‐derived CSF2 induces osimertinib resistance, whole‐transcriptome profiling of LUAD cells was compared between hIgG1‐Fc (control group) and recombinant CSF2‐Fc (rCSF2 group) treatment. This analysis revealed that both protein‐coding mRNAs and lncRNAs were differently expressed, and the differences were more apparent in terms of lncRNAs than mRNAs (Figure 4A; Figure S4). Of the 112 common differentially expressed lncRNAs in two cell lines (Figure 4B), we selected the top five upregulated lncRNAs after CSF2 exposure for further validation by qRT‐PCR, and the results showed that only lnc‐CSRNP3‐6:1 (briefly renamed as lnc‐CSRNP3) showed a marked increase in both cell lines (Figure 4C). Moreover, like CSF2, CAF^OR^‐CM also increased the expression level of lnc‐CSRNP3 in LUAD cells, which was attenuated by the anti‐CSF2 neutralizing antibody (Figure 4D). More importantly, CSF2 exposure significantly increased the IC_50_ values of osimertinib in LUAD cells, which could be dramatically reversed by lnc‐CSRNP3 knockout (Figure 4E). These data indicate that CAF^OR^‐derived CSF2 facilitates osimertinib resistance by promoting the expression of the lnc‐CSRNP3 in LUAD cells.

Next, we established a xenograft model of PC‐9 and CAF^OR^ co‐culture cells by subcutaneous injection in nude mice (Figure 4F). The anti‐CSF2 antibody or lnc‐CSRNP3 knockout significantly reduced the CSF2 content in mouse plasma and the lnc‐CSRNP3 expression in xenograft tissues, as well as markedly decreased the tumor volume and tumor weight (Figure 4G–L). In addition, the characteristics of xenograft tissues were also studied. The H&E staining demonstrated that the xenograft tumors exhibited both the increased size and abnormal arrangement of nuclei. Immunohistochemical staining exhibited that both anti‐CSF2 antibody and lnc‐CSRNP3 knockout reduced the expression of proliferation marker Ki‐67 while increasing the levels of cell cycle inhibitor P27^KIP1^ and cell apoptosis marker cle‐caspase3 (Figure 4M). Taken together, these data confirm the promoting roles of both CAF^OR^‐derived CSF2 and lnc‐CSRNP3 in osimertinib resistance in vivo.

### 2.5 CAF^OR^‐derived CSF2 upregulates lnc‐CSRNP3 through activating the JAK2/STAT3 pathway

**FIGURE 5:**
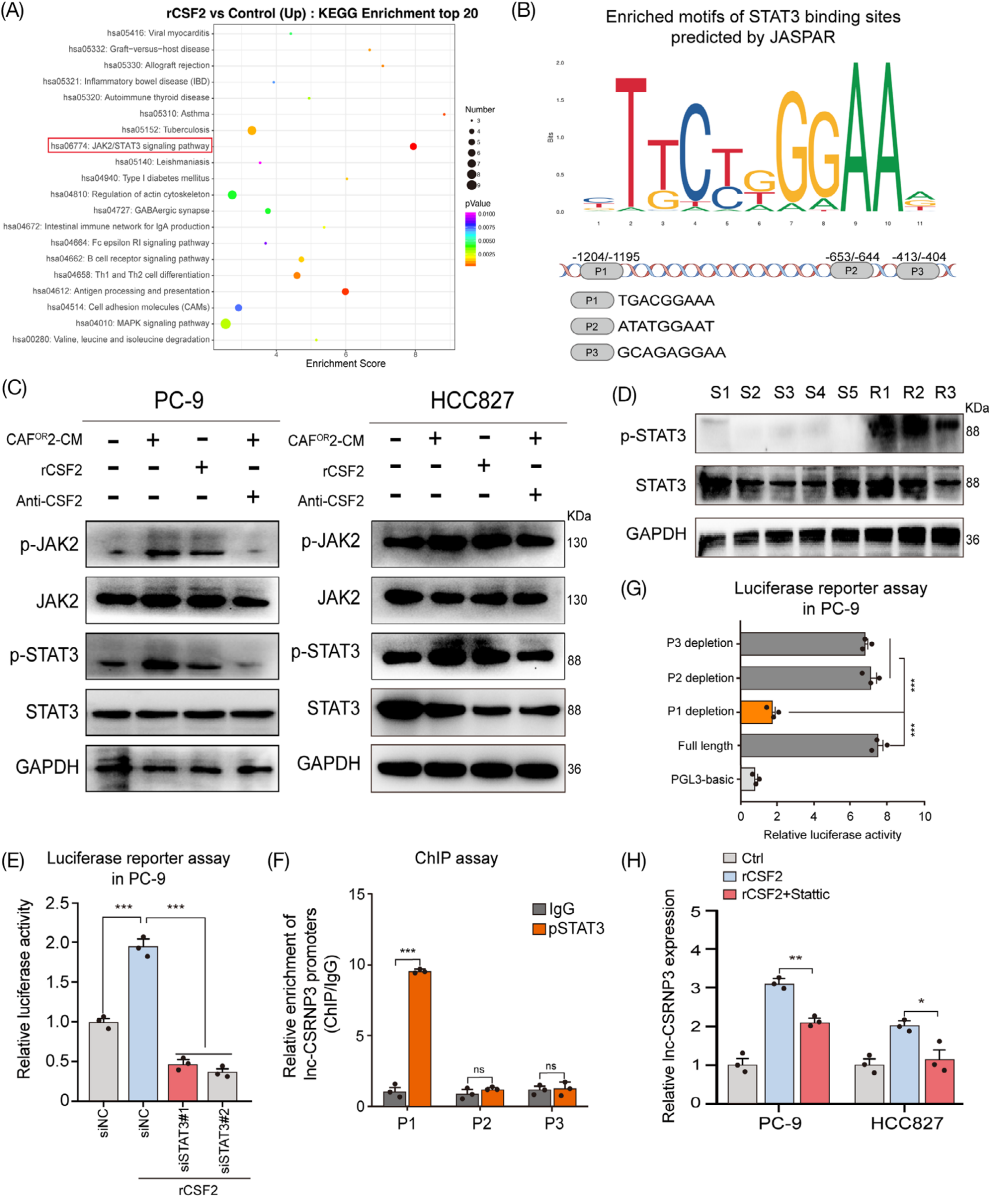
CAF^OR^‐derived CSF2 activated the JAK2/STAT3 signaling pathway to upregulate lnc‐CSRNP3. (A) The top 20 upregulated pathways were plotted based on the enriched gene ratio and *p*‐value in PC‐9 cells treated with rCSF2 compared with the control. (B) A conserved STAT3‐binding motif was predicted by JASPAR and schematic images of the potential STAT3 motif binding sites in the promoter of lnc‐CSRNP3 are shown. (C) Western blot analysis of JAK2, p‐JAK2, STAT3, and p‐STAT3 protein expression in PC‐9 and HCC827 cells treated with CAF^OR^2‐CM or rCSF2. A neutralizing antibody against CSF2 was used to deplete CSF2 in CAF^OR^2‐CM. (D) Western blot analysis of STAT3, and p‐STAT3 protein expression in LUAD tissues from osimertinib‐resistant and osimertinib‐sensitive patients. (E) Luciferase reporter assays of PC‐9 cells transfected with a reporter plasmid containing the lnc‐CSRNP3 promoter and treated with rCSF2 or STAT3 depletion. (F) ChIP analysis of the STAT3 occupancy at the promoter of lnc‐CSRNP3 in PC‐9 cells. (G) Luciferase reporter assays of PC‐9 cells treated with rCSF2 and transfected with reporter plasmids containing P1, P2, and P3 deletions in the lnc‐CSRNP3 promoter. (H) Lnc‐CSRNP3 expression in PC‐9 and HCC827 cells treated with rCSF2 (100 ng/mL) alone or rCSF2 and STAT3 inhibitor (Stattic, 2 µM) together for 3 days. The results are presented as the mean ± SD of three technical replicates, and each dot represents a technical replicate of the assay. *p < 0.05; **p < 0.01; ***p < 0.001; ns = no significance.

To further reveal the mechanism by which CAF^OR^‐derived CSF2 upregulates lnc‐CSRNP3 in LUAD cells, the KEGG pathway analysis was performed using differentially expressed mRNAs between the control and rCSF2 groups. Both the enrichment score and gene numbers indicated that the JAK2/STAT3 signaling pathway was enriched after CSF2 treatment (Figure 5A). Besides, GSEA analysis also showed these differentially expressed mRNAs were enriched in the JAK2/STAT3 signaling pathway (Figure S5A). Simultaneously, we analyzed the promoter region of lnc‐CSRNP3 and found three potential binding sites of STAT3 (Figure 5B). Treatment with CAF^OR^‐CM or CSF2 successfully activated the JAK2/STAT3 pathway in LUAD cells, which could be rescued by anti‐CSF2 antibodies (Figure 5C; Figure S5B). Additionally, the phosphorylated STAT3 was upregulated in osimertinib‐resistant LUAD tissues, but not in sensitive ones (Figure 5D). These results indicate that CAF^OR^‐derived CSF2 could activate the JAK2/STAT3 pathway in LUAD cells.

Then, we investigated whether STAT3 could directly bind to the promoter of lnc‐CSRNP3, and upregulate its expression. Luciferase reporter assays showed that CSF2 treatment significantly enhanced the transcriptional activity of the lnc‐CSRNP3 promoter in PC‐9 cells, which could be abrogated by STAT3 knockdown (Figure 5E). The chromatin immunoprecipitation (ChIP) assay further revealed that STAT3 directly bound to the fragment P1 of lnc‐CSRNP3 promoter (Figure 5F), and only the STAT3‐fragment P1 interaction could promote the transcription of lnc‐CSRNP3 in CSF2‐treated PC‐9 cells (Figure 5G). STAT3 inhibitor, Stattic, significantly reversed CSF2‐induced lnc‐CSRNP3 upregulation (Figure 5H).^25^ Altogether, these findings support that CAF^OR^‐derived CSF2 upregulates lnc‐CSRNP3 in LUAD cells by activating JAK2/STAT3 pathway.

### 2.6 Lnc‐CSRNP3 promotes osimertinib resistance by cis‐regulating nearby gene *CSRNP3*

**FIGURE 6:**
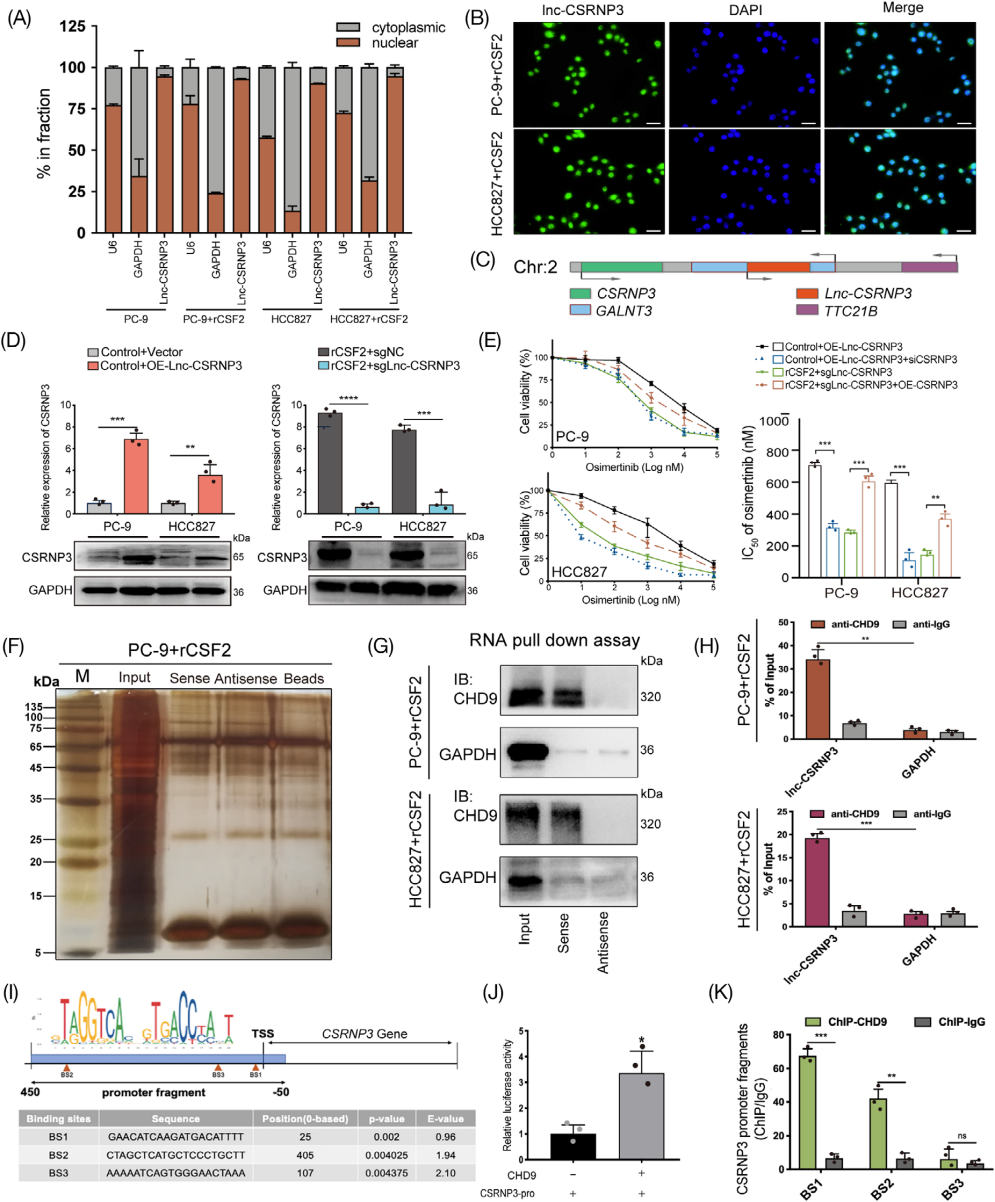
Lnc‐CSRNP3 promotes osimertinib resistance by cis‐regulating nearby gene *CSRNP3*. (A) The expression of lnc‐CSRNP3 in either the cytoplasm or nucleus of LUAD cell lines. (B) FISH assays identifying the subcellular location of lnc‐CSRNP3 in LUAD cells cultured with rCSF2 (100 ng/mL). Scale bar = 100 µm. (C) Schematic diagram of lnc‐CSRNP3 genomic nearby genes. (D) The influence of lnc‐CSRNP3 on the expression of nearby gene *CSRNP3*. (E) The influence of lnc‐CSRNP3 on osimertinib sensitivity was rescued by CSRNP3. (F) Silver staining of lnc‐CSRNP3 pull‐down proteins in PC‐9 cells cultured with rCSF2 (100 ng/mL). (G) CHD9 was pulled down by biotin‐labeled sense lnc‐CSRNP3 but not lnc‐CSRNP3 antisense RNA in the indicated cells. (H) RIP assays were applied using anti‐CHD9 antibodies with extractions from PC‐9 or HCC827 cells cultured with rCSF2 (100 ng/mL). (I) JASPR website predicted the specific binding site (BS) of CHD9 with the *CSRNP3* promoter region. (J) Double fluorescent luciferase report assay showed that CHD9 promoted *CSRNP3* gene transcription in HEK293 cells. (K) ChIP‐qPCR assay showed that CHD9 primarily bands with BS1 and BS2 in the *CSRNP3* promoter region. The results are presented as the mean ± SD of three technical replicates, and each dot represents a technical replicate of the assay. *p < 0.05; **p < 0.01; ***p < 0.001; ****p < 0.0001, ns = no significance.

To elucidate the potential molecular mechanisms through which lnc‐CSRNP3 promotes osimertinib resistance, we first observed its subcellular localization in LUAD cells, because the functional mechanisms of lncRNAs are dependent on their subcellular distribution.^26^ Our results showed that lnc‐CSRNP3 was prevailingly localized in the nuclear compartment (Figure 6A, B). Given that regulating the expression of nearby genes is one of the important ways in which the nuclear lncRNAs exert their biological effects,^27^ we observed the chromatin localization of lnc‐CSRNP3 and found that it was in the vicinity of three protein‐coding genes, UDP‐GalNAc transferase 3 (*GALNT3*), cysteine/serine‐rich nuclear protein 3 (*CSRNP3*) and tetratricopeptide repeat protein 21B (*TTC21B*) (Figure 6C). Among them, only the level of CSRNP3 could be significantly modulated by lnc‐CSRNP3 in LUAD cells (Figure 6D; Figure S6). Moreover, the results of the CCK‐8 assay showed that lnc‐CSRNP3‐induced decrease in osimertinib sensitivity could be rescued by *CSRNP3* knockdown (Figure 6E), indicating that lnc‐CSRNP3 promotes osimertinib resistance by upregulating CSRNP3 expression.

To investigate the process by which lnc‐CSRNP3 controls the expression of CSRNP3, we utilized a biotin‐labeled RNA pulldown experiment, followed by mass spectrometric analysis, to identify the proteins that potentially interact with lnc‐CSRNP3. (Figure 6F). Our results revealed that chromodomain helicase DNA binding protein 9 (CHD9) was the highest‐ranked interactor. Then, the interaction of lnc‐CSRNP3 with CHD9 was confirmed via biotin‐labeled RNA pull‐down along with western blotting assays and anti‐CHD9 RIP assays (Figure 6G, H). According to previous studies, CHD9 exerts its biological effects by directly binding to the promoter of downstream genes and acts as transcriptional coactivator or chromatin remodeling factor.^28^ Remarkably, we have discovered three CHD9 binding sites in the promoter region of CSRNP3 using the JASPAR database. (Figure 6I). Then we performed dual luciferase assay and anti‐CHD9 ChIP assay, confirming that CHD9 bound directly to the BS1 and BS2 fragments of *CSRNP3* promoter and promoted its transcription (Figure 6J, K). Taken together, these observations suggest that lnc‐CSRNP3 enhances CSRNP3 expression through directly interacting with CHD9.

### 2.7 CSRNP3 promotes ribosome biosynthesis by directly binding to PP1α

**FIGURE 7:**
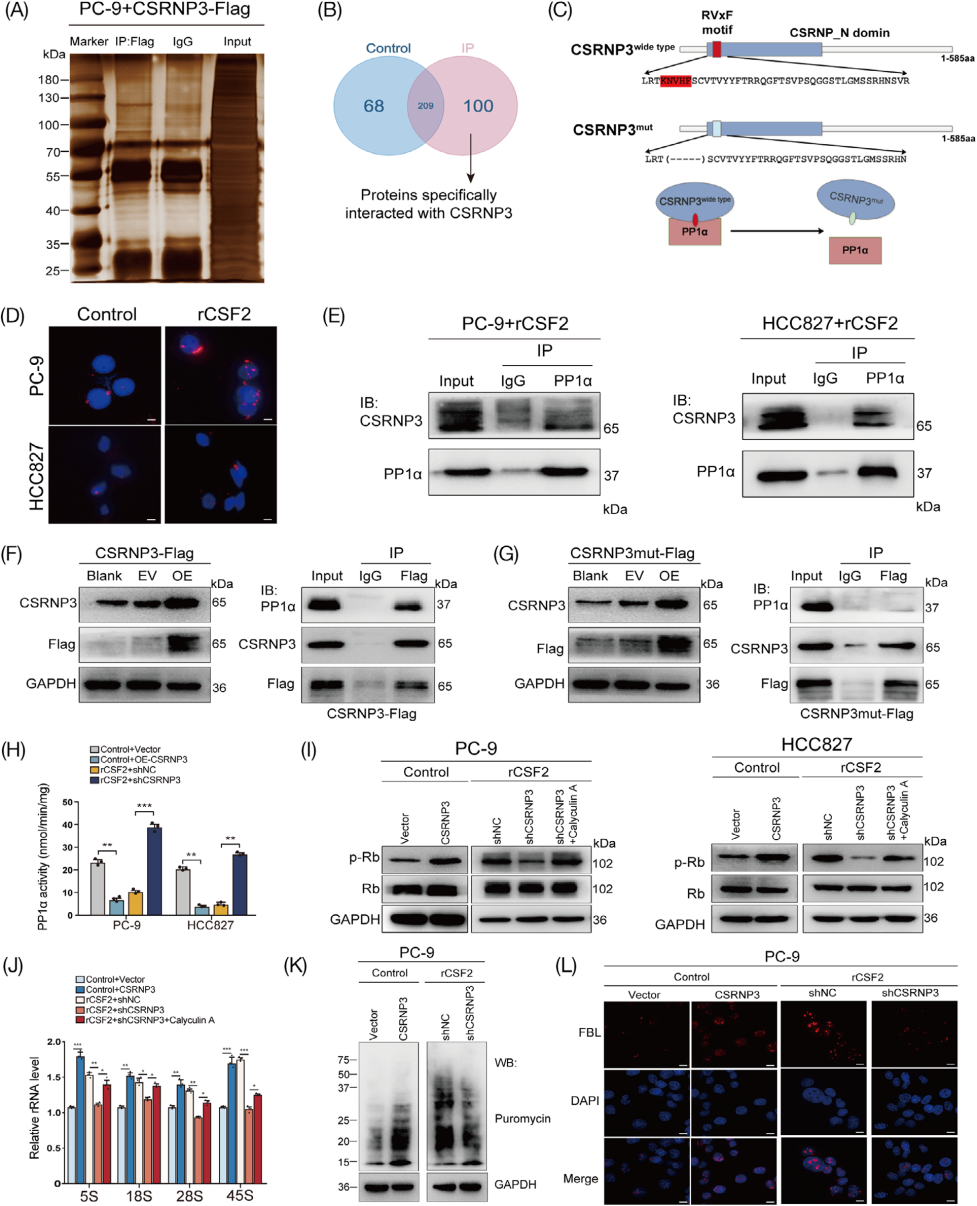
CSRNP3 directly binds to PP1α and promotes ribosome biosynthesis. (A) Silver staining of CSRNP3 interacting proteins. IP assays were performed using anti‐Flag antibodies in PC‐9 cells transfected with a vector expressing the Flag‐CSRNP3. (B) Venn diagram showed the number of differentially CSRNP3‐interacted proteins between control (anti‐IgG) and IP (anti‐Flag). (C) Schematic diagram of CSRNP3 and PP1α protein‐protein interaction according to BioGRAD database. (D) PLA assays were used to determine the endogenous interaction between CSRNP3 and PP1α. Scale bar = 20 µm. (E) Endogenous interaction between CSRNP3 and PP1α was determined by Co‐IP assays using anti‐PP1α in PC‐9 or HCC827 cells cultured with rCSF2 (100 ng/mL). (F, G) Overexpression of CSRNP3‐Flag or CSRNP3mut‐Flag in PC‐9 cells and Semi‐endogenous interaction between CSRNP3 and PP1α was determined by Co‐IP assays using anti‐Flag in the indicated cells. (H) The phosphatase activity kit showed CSRNP3 inhibited PP1α activity in LUAD cells. (I) Knockdown CSRNP3 decreased p‐Rb protein levels, which could be abolished by PP1α inhibitor, calyculin A (25 nM). (J) Knockdown of CSRNP3 decreased rRNA expression, which could be abolished by calyculin A. (K) Puromycin incorporation assay showed CSRNP3 promoted protein synthesis in PC‐9 cells. (L) Immunofluorescence of fibrillarin (FBL) showed CSRNP3 promoted ribosome biogenesis. Scale bar = 20 µm. The results are presented as the mean ± SD of three technical replicates, and each dot represents a technical replicate of the assay. *p < 0.05; **p < 0.01; ***p < 0.001; ****p < 0.0001, ns = no significance.

To further explore the downstream molecules regulated by CSRNP3, we used mass spectrometry to identify its potential interacting proteins in LUAD cells (Figure 7A). As shown in Figure 7B, a total of 100 proteins were screened out. Among them, 51 proteins were co‐located in the nucleus with CSRNP3, including the catalytic subunit of Ser/Thr phosphatase‐1 (PP1) (Figure S7A, B, Table S3). A previous study has shown that CSRNP3 contains an RVxF motif, which binds to the backside of PP1.^29^ In addition, using the BioGRAD database (https://thebiogrid.org/), we found that there might exist a protein–protein interaction (PPI) between CSRNP3 and a PP1 catalytic subunit, PP1α (Figure 7C). Therefore, we performed proximity ligation assay (PLA) and co‐immunoprecipitation (Co‐IP) assays to investigate their interaction, and found that CSRNP3 indeed directly interacted with PP1α through its RVxF motif (Figure 7D–G).

PP1α, which belongs to the phosphoprotein phosphatase family, is a ubiquitous eukaryotic enzyme that dephosphorylates a broad range of phosphoproteins,^30^ and its interacting proteins could change its expression, subcellular distribution or directly inhibit its enzyme activity.^31^ Consistent with previous results, we found that the binding of PP1α by CSRNP3 inhibited PP1α activity, but had no effect on its expression or subcellular distribution in NSCLC cells (Figure 7H; Figure S7C, D). As a phosphatase, PP1α has many nuclear substrates such as phosphorylated mouse double minute 2, myocyte enhancer factor 2A, retinoblastoma (p‐Rb), and cAMP‐response element binding protein, playing a pivotal role in tumor progression.^29^ To determine the downstream molecules of the CSRNP3/PP1α axis, we performed a western blotting assay and found that the protein levels of p‐Rb were dramatically upregulated after CSRNP3 overexpression in LUAD cells, while were downregulated after CSRNP3 knockdown, which could be abolished by PP1α inhibitor, calyculin A (Figure 7I).^32^ According to previous research, p‐Rb was involved in ribosome biosynthesis by promoting ribosomal RNA (rRNA) transcription.^33^, ^34^, ^35^ By using qRT‐PCR, we found that the levels of 5S, 18S, 28S, and 45S rRNA transcripts were markedly increased after CSRNP3 overexpression in PC‐9 cells, but were decreased after CSRNP3 knockdown, which could be rescued by calyculin A (Figure 7J). Puromycin incorporation assays demonstrated that CSRNP3 could promote protein synthesis in PC‐9 cells (Figure 7K). Furthermore, immunofluorescence of fibrillarin (FBL), which is participated in pre‐rRNA processing and used as a ribosome biosynthesis marker,^36^ showed that CSRNP3 could significantly enhance the ribosome biosynthesis (Figure 7L). Altogether, these data demonstrate that CSRNP3 can promote ribosome biosynthesis through directly binding to PP1α.

### 2.8 Inhibition of CSF2 pathway overcomes osimertinib resistance in LUAD PDX mice model

**FIGURE 8:**
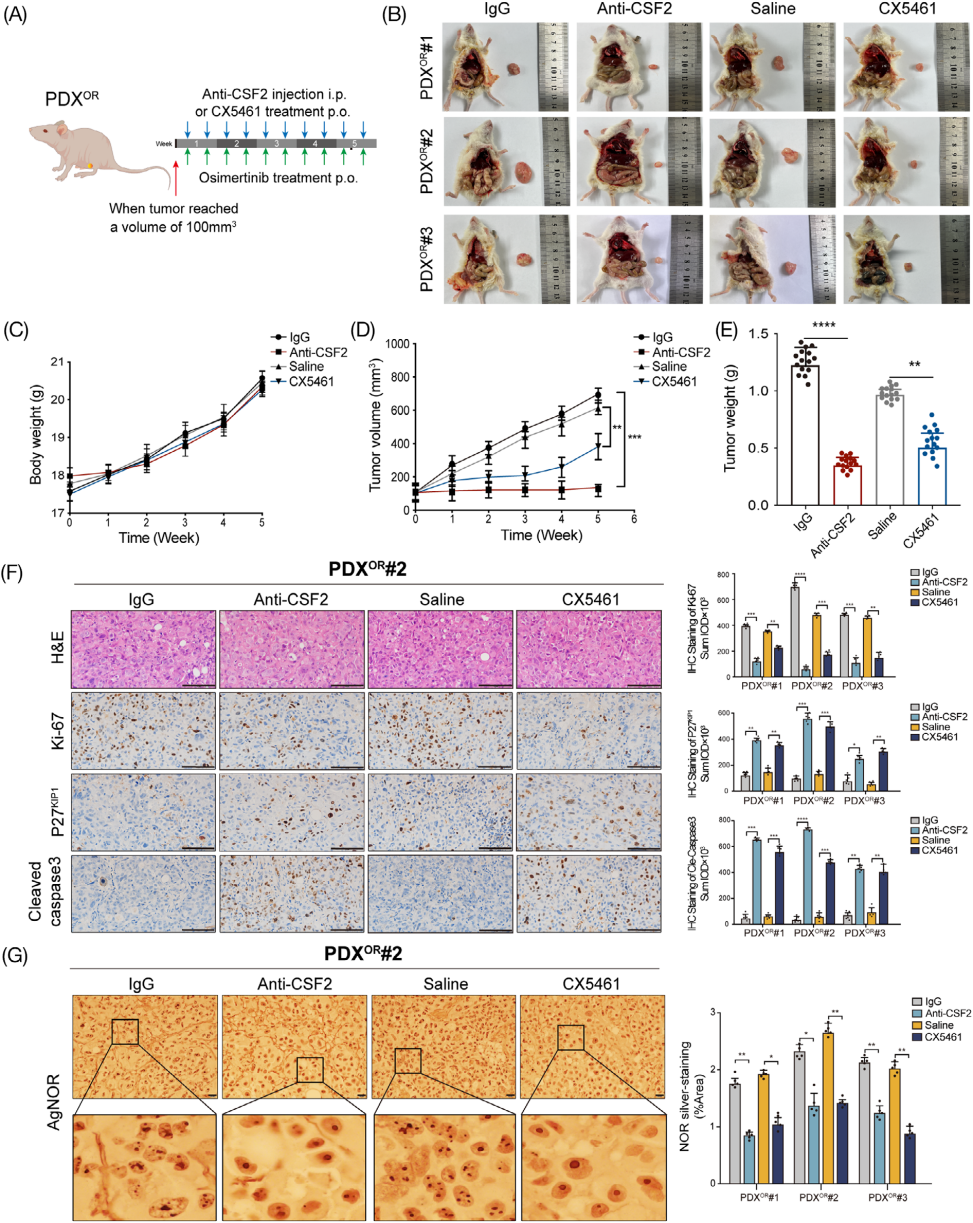
Targeting lnc‐CSRNP3 or ribosomal biosynthesis overcame resistance to osimertinib in vivo. (A) Once the tumors reached 100^3^ mm in volume, PDX^OR^ model mice receiving an lnc‐CSRNP3 antisense oligo (ASO, 10 nM, i.v.) or rRNA synthesis inhibitor CX5461 (50 mg/kg, p.o.), and osimertinib (10 mg/kg, p.o.) twice a week. (B) Representative image of tumors from different groups in PDX^OR^ model mice (*n *= 5 mice/group). (C) Average body weight curves of PDX^OR^ model mice in different groups (*n *= 5 mice/group). (D‐E) Tumor growth of PDX^OR^ model mice in different groups (*n *= 5 mice/group). (F, G) H&E staining in xenograft tumors. Scale bar = 100 µm; *n *= 5 mice/group. Representative immunohistochemical staining of Ki‐67, P27^KIP1^, cle‐caspase3 in PDX^OR^#2 xenograft tumors (scale bar = 100 µm) and AgNOR staining in PDX^OR^#2 xenograft tumors. Scale bar = 20 µm; *n *= 5 mice/group. *p < 0.05; **p < 0.01; ***p < 0.001; ****p < 0.0001, ns = no significance.

Finally, we sought to validate the function of the CSF2 pathway in vivo and intended to find potential therapeutic strategies for osimertinib resistance in LUAD. For these purposes, we generated osimertinib‐resistant PDX models (PDX^OR^) (Figure S8) and observed the therapeutic efficacy of anti‐CSF2 neutralizing antibody and ribosome biogenesis inhibitor CX5461 against osimertinib resistance (Figure 8A). Our findings indicate that by targeting CSF2 or ribosome biogenesis, it is possible to drastically reduce both the volume and weight of tumors. However, mice in various groups had similar average body weights (Figure 8B–E). In addition, both anti‐CSF2 and CX5461 decreased the expression of Ki‐67 but increased the level of P27^KIP1^ and cle‐caspase3 (Figure 8F). According to previous reports, a greater number of AgNOR dots per nucleus and a proportion of NOR‐occupied nuclear area indicate more active ribosome biogenesis.^30^ Our results showed that both the AgNOR dots number per nucleus and NOR sliver‐staining area were significantly decreased after targeting lnc‐CSRNP3 or ribosome biogenesis (Figure 8G). These results suggest that targeting the CSF2 pathway can efficiently overcome the resistance to osimertinib in vivo.

## 3 DISCUSSION

Osimertinib, a third‐generation EGFR‐TKI, is currently indicated as first‐line therapy in LUAD patients with sensitizing EGFR mutations. Acquired resistance in osimertinib is the main cause of treatment failure in LUAD, and strategies for overcoming this phenomenon are critical for improving therapeutic efficacy. To date, many investigations have identified resistance mechanisms using bulk tumor tissues or subcloned resistant cell lines, including EGFR secondary mutation, the activation of alternative signaling pathways, or phenotypic transformation of tumor cells.^37^, ^38^ Nevertheless, the presence of intratumoral heterogeneity might lead to varying reactions to chemotherapies, necessitating supplementary methods to uncover the whole range of resistance mechanisms.^39^ In this study, by using snRNA‐seq, we found that CAFs in tumor microenvironment were essential for osimertinib resistance. This conclusion is consistent with recent research indicating that the interplay between CAFs and lung cancer cells may play an important role in osimertinib resistance. For example, CAFs have been proven to secrete many cytokines or growth factors, such as IL‐6, periostin, hepatocyte growth factors, and insulin‐like growth factors, mediating an osimertinib‐resistant phenotype in lung cancer cells.^40^, ^41^, ^42^, ^43^, ^44^ However, these studies have not conducted in‐depth research about CAF subpopulation, neglecting the heterogeneity of CAFs. Thus, the roles and underlying mechanisms of precise CAF subpopulation in the development of acquired osimertinib resistance in LUAD still remain to be elucidated.

Although CAFs play a crucial role in osimertinib resistance, the specific subset of CAFs that contributes to this resistance is not well characterized. In this study, we observed that CAF^OR^s were mainly characterized as iCAFs which could induce osimertinib resistance in LUAD cells. Consistent with our findings, the iCAF phenotype might also possess tumor‐promoting properties and confer chemoresistance to pancreatic ductal adenocarcinoma.^45^ However, the biological function and underlying mechanisms of iCAFs in LUAD remain poorly understood. As far as we know, this is the initial publication that offers understanding of the interaction between iCAFs and LUAD cells in the formation of osimertinib resistance. Then, we demonstrated that CSF2 was required for CAF^OR^‐induced osimertinib resistance. Current studies have found that CSF2 is mainly secreted by macrophages, T cells, endothelial cells, fibroblasts, and tumor cells.^18^, ^46^ In our work, we found that increased CSF2 after osimertinib resistance was predominantly secreted by CAFs but not tumor cells. Considering that the CAFs are the most abundant mesenchymal cells in the tumor microenvironment, we supposed that CAFs‐derived cytokines might become an ideal biomarker for osimertinib resistance in LUAD. Consistent with this hypothesis, we found the plasma CSF2 level in osimertinib‐resistant LUAD patients was higher than that in sensitive ones, suggesting that the plasma level of CSF2 may be a potential marker to monitor osimertinib resistance in LUAD.

Subsequently, we observed that long noncoding RNA (lncRNA), *lnc‐CSRNP3*, was upregulated by CSF2/JAK2/STAT3 signal and contributed to the development of osimertinib resistance. LncRNAs are transcripts with a length of more than 200 nucleotides but no protein‐coding capacity.^47^ In recent years, more and more studies have suggested that lncRNAs are involved in acquired resistance to osimertinib in LUAD through various mechanisms. To cite a few, lncRNA HIF1A‐AS2 promotes osimertinib resistance by activating the IL‐6/STAT3 pathway,^48^ lncRNA CRNDE induces osimertinib resistance through inhibiting the eukaryotic translation initiation factor 4A3 (eIF4A3)/mucin 1 (MUC1)/EGFR pathway.^49^ As far as we know, this report is the first to offer understanding of how lnc‐CSRNP3 functions in causing osimertinib resistance. Our discoveries highlight that the CSF2/lnc‐CSRNP3 relationship could be a new target for treatment to overcome osimertinib resistance in lung cancer patients.

Given that lnc‐CSRNP3 is mainly located in nuclear and could cis‐regulate the neighboring genes, we found its downstream molecule, *CSRNP3*. As a member of the CSRNP family, just a few studies have examined the biological function of CSRNP3. Current research reveals that it is closely related to muscle development, obesity, metabolic syndrome, and also serves as a prognostic biomarker in clear cell renal cell carcinoma,^50^, ^51^, ^52^ but its function in EGFR‐TKIs resistance is unknown. Prior studies have shown that differential methylation of promoters and alteration of histones play a role in the expression of the mouse *Csrnp‐3* gene.^53^ In this study, we found that lnc‐CSRNP3 modulated the expression of CSRNP3 through binding with CHD9. CHD9 is an ATP‐dependent chromatin remodeling enzyme that belongs to the chromodomain‐helicase‐DNA‐binding (CHD) family and has well‐documented roles in transcription regulation.^54^ For example, CHD9 upregulates RUNX2 and has a potential role in skeletal evolution.^55^ In addition, CHD9 binds to skeletal tissue‐specific promoters and participates in the transcriptional regulation of osteogenic cells’ maturation.^56^ Our work shows for the first time that CHD9 mediates the positive regulation between lnc‐CSRNP3 and CSRNP3.

Finally, we found CSRNP3 directly interacted with PP1α through its “RVxF” motif, indicating that CSRNP3 was a PP1‐interacting protein (PIP).^29^ Recent research about PIPs has shown that some PIPs such as protein phosphates 1 nuclear‐targeting subunit can form a stable complex with PP1, and guide its phosphatase activity, subcellular distribution or substrate specificity, playing an important role in cancer development.^57^ In this study, we found that CSRNP3 could inhibit PP1α phosphatase activity, and modulate the phosphorylation levels of p‐Rb. It is widely acknowledged that p‐Rb takes part in cell cycle progression, senescence, and proliferation.^58^, ^59^ Moreover, Rb is associated with ribosomal biogenesis by regulating rRNA transcription.^33^ consistently, we also found that ribosomal biogenesis could be modulated by the CSRNP3/PP1α/p‐Rb complex, indicating the crucial role of ribosomal biosynthesis in the development of acquired resistance to osimertinib in LUAD. Furthermore, CX5461, a rRNA synthesis inhibitor that could target RNA polymerase I and reduce solid tumor development, is now under a phase I clinical trial in patients with advanced hematologic cancers and shows predictable pharmacokinetics and a safety profile allowing prolonged dosing.^60^ In this study, we found that CX5461 also could overcome the resistance to osimertinib in osimertinib‐resistant PDX models. Therefore, this study provided preclinical support for using CX5461 for the treatment of osimertinib‐resistant LUAD cells.

One of our study's drawbacks is the difficulty of obtaining paired tissue samples from osimertinib‐sensitive and osimertinib‐resistant LUAD patients, which prevents us from using enough samples for target identification and validation. As a result, it is almost impossible to exclude the interindividual differences in cellular components of TME. Second, owing to the low success rate and long modeling period of the LUAD PDX model, only three PDX models have been successfully established so far, and the efficacy of CSF2 antibody or CX5461 to overcome osimertinib resistance is still in the early research phase. Finally, although iCAFs have been indicated to play an important role in acquired resistance to osimertinib in this study, iCAFs in CAF^OR^ have not been purified, leading to the possible involvement of other CAF subsets in our findings.

**FIGURE 9:**
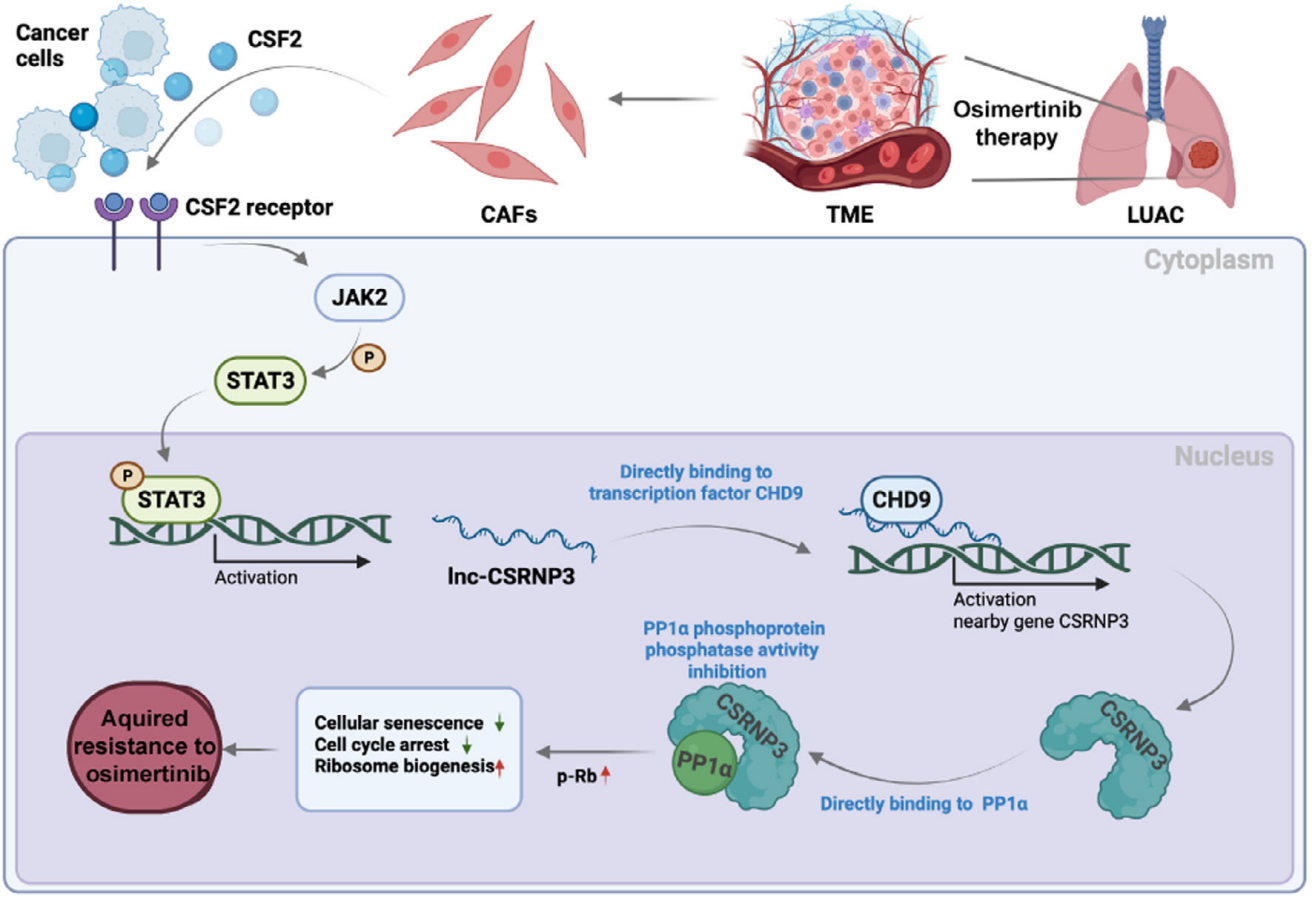
Illustration of CAF^OR^‐derived CSF2 mediating lnc‐CSRNP3 activation in LUAD cells to induce osimertinib resistance. CAF^OR^‐derived CSF2 was essential for osimertinib resistance by modulating the JAK2/STAT3/lnc‐CSRNP3 axis. Lnc‐CSRNP3 directly binds to CHD9, promotes *CSRNP3* gene expression, and inhibits the phosphatase activity of PP1α, thereby inducing osimertinib resistance by enhancing ribosome biogenesis.

In summary, we found that CAF^OR^‐derived CSF2 modulates the JAK2/STAT3/lnc‐CSRNP3 axis, which increases ribosome biogenesis in LUAD cells and leads to resistance to osimertinib treatment (Figure 9). In this case, CSF2 is crucial to CAF‐LUAD cell communication, underscoring the need for reasonable therapies that target the CSF2/lnc‐CSRNP3 axis to overcome osimertinib resistance in LUAD patients.

## 4 MATERIALS AND METHODS

### 4.1 Cell lines

HEK293 cells and human LUAD cell lines PC‐9 and HCC827(EGFR19del) were obtained from the National Collection of Authenticated Cell Culture of China. The cells were grown in RPMI 1640 medium (Gibco) with 10% fetal bovine serum at 37°C in a humidified environment with 5% CO_2_ and 95% air. All cell lines were mycoplasma‐screened and verified by STR profiling.

### 4.2 Ethics statement

This study was approved by the Ethics Committee Board of Chongqing Medical University (no. 2017009) and was registered with the Chinese Clinical Trial Registry on January 27, 2018 (no. ChiCTR1800014660). A total of 30 LUAD patients who underwent first‐line osimertinib therapy (15 osimertinib‐sensitive patients and 15 acquired osimertinib‐resistant patients) were enrolled. Written informed consent was obtained from all of them. Details of patients are listed in Table S4.

### 4.3 RNA extraction and quantitative real‐time PCR

Total RNA was extracted from whole‐cell lysate using TRIzol. The miRNeasy Serum/Plasma Kit (QIAGEN) extracted total RNA from clinical plasma samples. A PARIS kit (Thermo‐life) separated nuclear and cytoplasmic RNA, and the percentages in fractions were calculated using the formula: 2^Ct(nuclear)^/2^Ct (nuclear)^ + 2^Ct (cytoplasm)^ or 2^Ct(cytoplasm)^/ 2^Ct (nuclear)^ + 2^Ct (cytoplasm)^. Then, RNA was reverse transcribed into cDNA using the PrimeScript RT Reagent Kit (Takara Bio). qRT‐PCR was performed using SYBR Premix Ex Taq II (Takara Bio) according to the manufacturer's instructions. The primer sequences used in this study are shown in Table S5.

### 4.4 Proteins purification and western blotting assay

Total protein was extracted and quantified with the Pierce BCA protein assay kit (Thermo Fisher Scientific). Transferring proteins to PVDF membranes following 10% SDS‐PAGE. After 1 h of blocking with 5% BSA, the membrane was incubated with primary antibodies overnight at 4°C (Table S6). After 1 h of incubation with HRP‐conjugated secondary antibody, immunoreactive bands were identified using ECLTM Prime (GE Healthcare) and LAS‐3000 imager.

### 4.5 Lentivirus transfection and stable cell line construction

Lentiviruses overexpressing lnc‐CSRNP3 or CSRNP3 and the CRISPR/Cas9 system targeting it were transfected into LUAD cells for 48 h with 5 mg/mL polybrene. Stable cell clones were selected for 1 week with puromycin (5 µg/mL). The overexpression or knockdown efficiency was measured by qRT‐PCR. The sequences of siRNA, shRNA, and gRNA used are provided in Table S7.

### 4.6 ChIP assay

Following manufacturer instructions, Pierce Magnetic ChIP kit (Thermo Fisher Scientific) was used for the ChIP experiment. Approximately 4 × 10^6^ cells were collected and crosslinked in 1% formaldehyde. Next, MNase (ChIP grade) and sonication produced 200−1000 bp DNA fragments. The antibody‐target protein‐DNA complex was formed by adding anti‐CHD9 or anti‐STAT3 (Abcam) and immunoprecipitating it with Protein A/G magnetic beads. Washing and reversing cross‐links purified the concentrated DNA for qRT‐PCR analysis. The primer sequences are listed in Table S8.

### 4.7 Statistical analysis

The statistical analyses were done using SPSS 20.0 (IBM) and GraphPad Prism 6.0. Data were validated in three different experiments and presented as means ± SD, with comparable variance among groups. A one‐way ANOVA and two‐tailed Student's *t*‐test were used to compare data sets. A *p*‐value < 0.05 indicated statistical significance.

## AUTHOR CONTRIBUTIONS

Yu‐tang Huang investigated the study, administered the project, and wrote the original draft. Xiao‐Qing Wang administered the project. Chun‐Jie Wen validated and visualized the study. Jing‐chan Wang investigated the study. Hong‐Hao Zhou supervised the study. Lan‐Xiang Wu reviewed and edited the manuscript, visualized the study, and acquired the funding. All authors have read and approved the final manuscript.

## CONFLICT OF INTEREST STATEMENT

The authors declare no conflict of interest.

## ETHICS STATEMENT

This study was approved by the Ethics Committee Board of Chongqing Medical University (no. 2017009) and was registered with the Chinese Clinical Trial Registry on January 27, 2018 (no. ChiCTR1800014660). Written informed consent was obtained from all participants.

## Supporting information

Supporting Information

Supporting Information

## Data Availability

Raw sequencing data reported in this paper have been deposited at the Genome Sequence Archive at the National Genomics Data Center (Beijing, China) under the BioProject ID PRJCA027382. All data included in this study are available from the corresponding author upon a reasonable request.
