# Supplementary material for: Cancer‐associated fibroblast‐derived colony‐stimulating factor 2 confers acquired osimertinib resistance in lung adenocarcinoma via promoting ribosome biosynthesis
Source: MedComm (2020). 2024 Jul 20;5(8):e653. doi: 10.1002/mco2.653 (PMC11260172; doi:10.1002/mco2.653)
Supplement: Supplementary file 1 — Supporting Information [file MCO2-5-e653-s001.docx]

**Cancer-associated fibroblast-derived colony-stimulating factor 2 confers acquired osimertinib resistance in lung adenocarcinoma via promoting ribosome** **biosynthesis**

Yutang Huang^1^, Xiaoqing Wang^1^, Chunjie Wen^1^, Jingchan Wang^2^, Honghao Zhou^1,3^, Lanxiang Wu^1*^

^1^Institute of Life Sciences, Chongqing Medical University, Chongqing 400016, China

^2^School of Stomatology, Chongqing Medical University, Chongqing 400016, China

^3^Pharmacogenetics Research Institute, Institute of Clinical Pharmacology, Central South University, Changsha 410078, China

^*^Lead contact and to whom correspondence should be addressed:

Lanxiang Wu, PhD

Professor

Institute of Life Sciences, Chongqing Medical University

Email: lxwu@cqmu.edu.cn

**Supplementary figures**

Figure S1

**
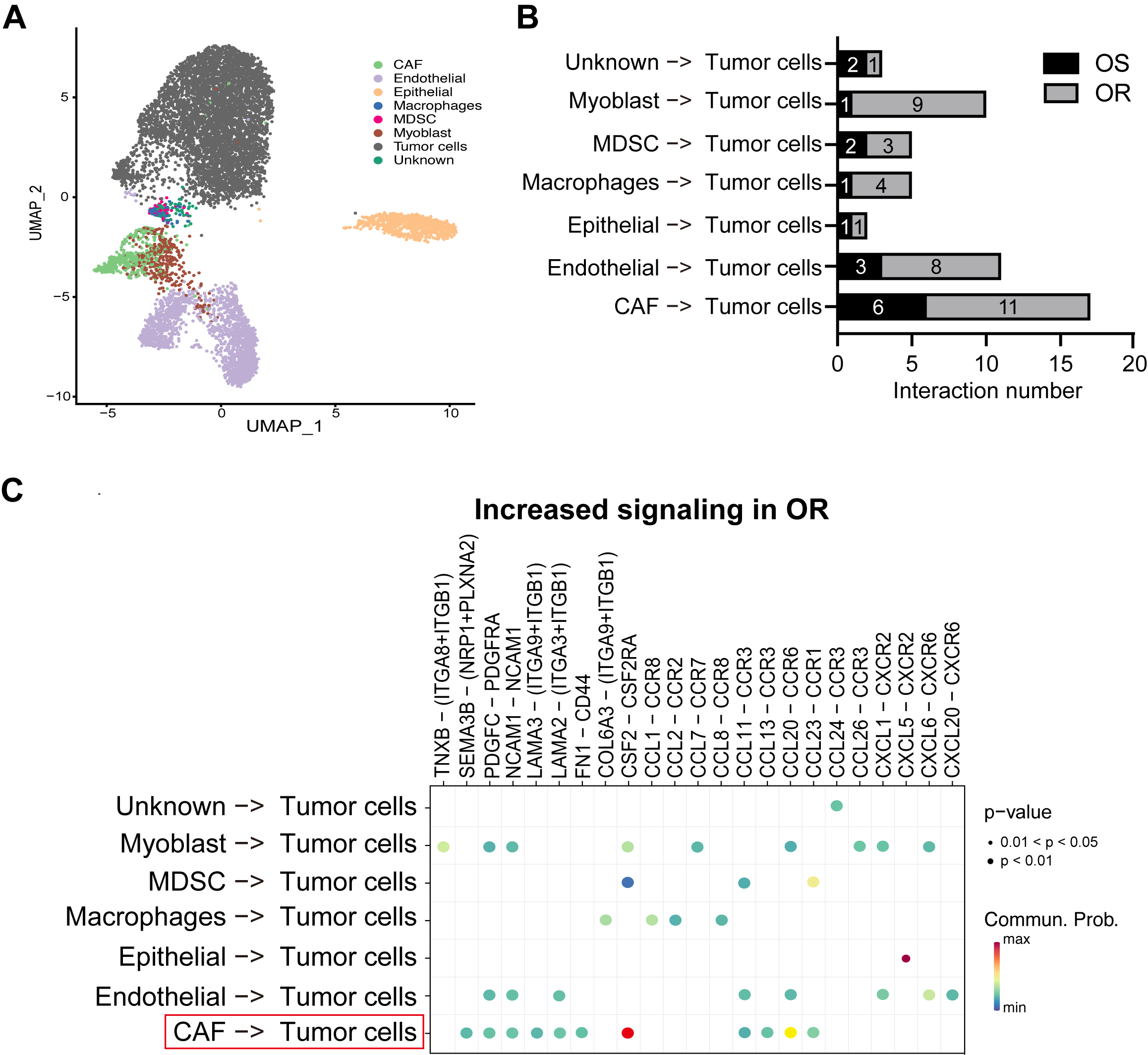
**

**Fig. S1** Cell chat analysis in new cell type. **(A)** Uniform manifold approximation and projection (UMAP) plots displaying 12,061 cell profiles with each cell color-coded for associated new cell type. **(B)** Interaction number between new cell type and tumor cells in OS and OR samples. **(C)** Bubble plot of increased signaling in OR.

Figure S2

**
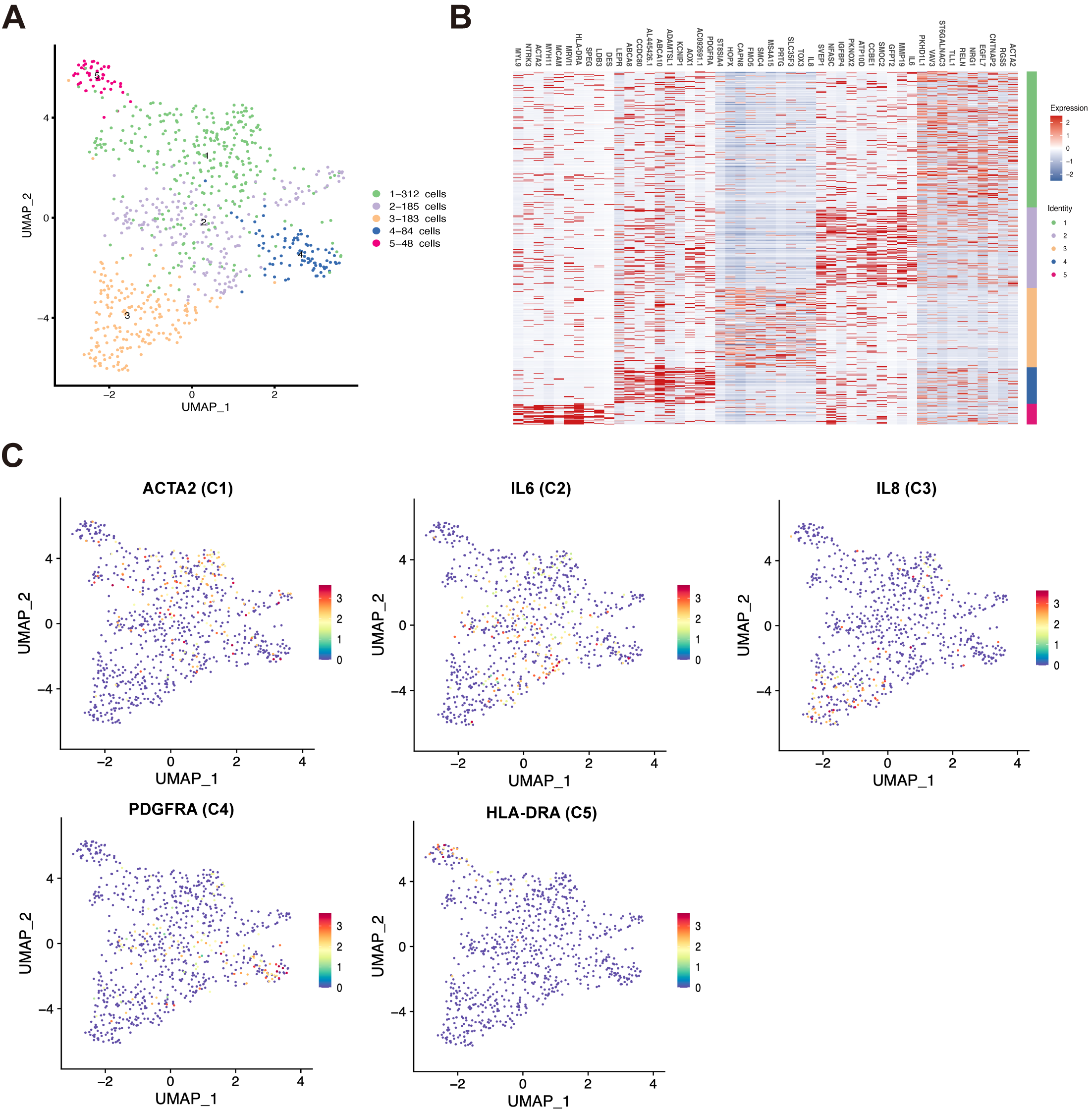
**

**Fig. S2** Single nucleus RNA sequencing (snRNA) proﬁles of ﬁbroblast cell lineages. **(A)** Re-clustering of 812 ﬁbroblast cells color-coded by clusters. **(B)** Heatmap shows differentially expressed marker genes in ﬁve sub-clusters. Red and blue: high and low expression, respectively. **(C)** Uniform manifold approximation and projection (UMAP) plots for marker gene expression for ACTA2 (Cluster 1, C1), IL6 (C2), IL8 (C3), PDGFRA (C4), HLA-DRA (C5).

Figure S3


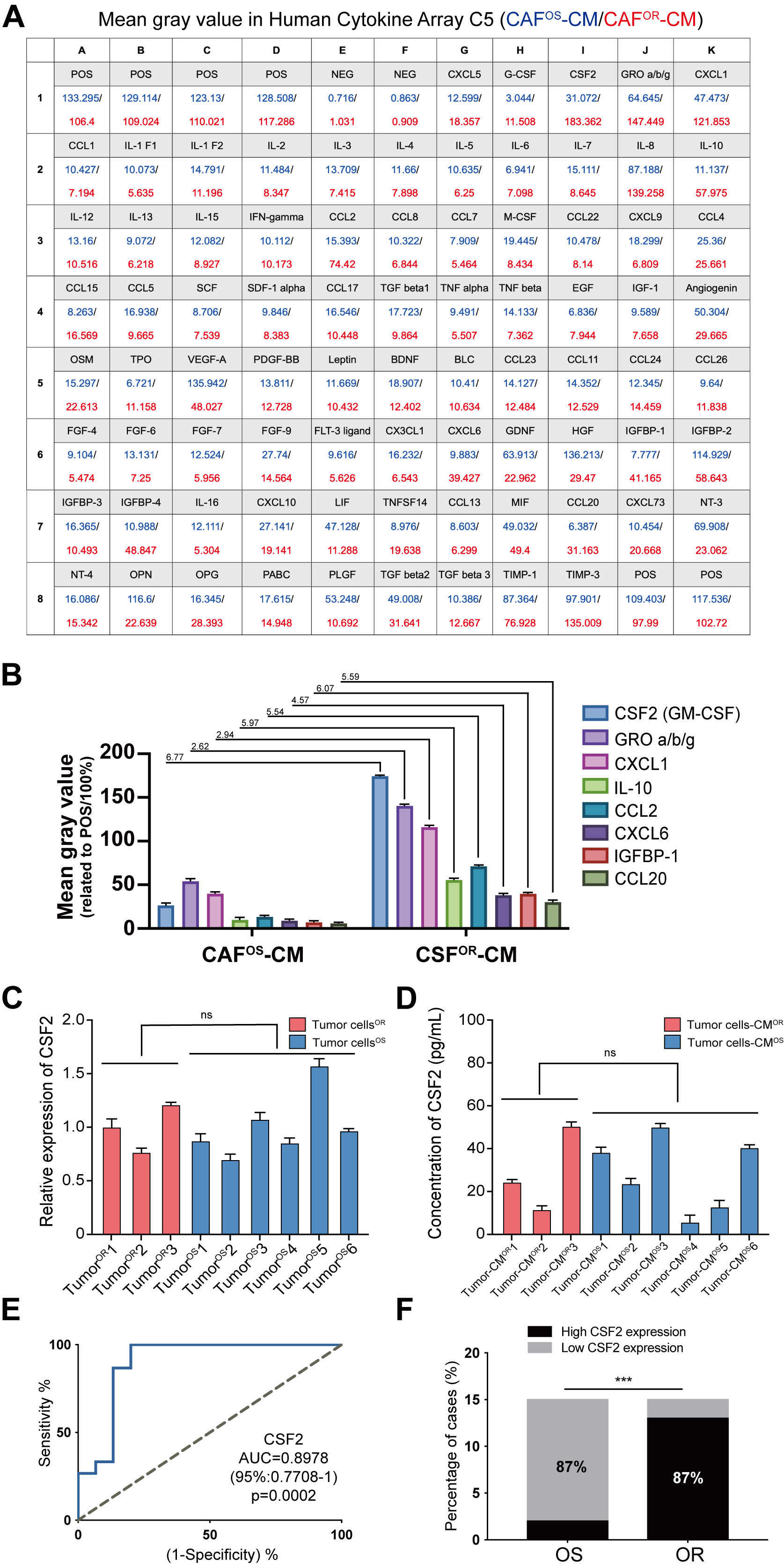


**Fig. S3** CSF2 was enriched in CAF^OR^-CM and correlated with osimertinib resistance. **(A)** The mean gray value of cytokine spots on the cytokine array membranes shown in Fig. 3A were assessed by ImageJ software (blue indicated CAF^OS^-CM; red indicated CAF^OR^-CM). **(B)** The fold change of eight upregulated cytokines in CAF^OR^-CM compared with CAF^OS^-CM (the mean gray values were normalized between membranes by setting the gray values of reference spots to 100%). **(C)** The expression of CSF2 in the osimertinib-sensitive and -resistant primary tumor cells was examined by qRT-PCR. **(D)** The expression of CSF2 in the culture medium of osimertinib-sensitive and -resistant primary tumor cells was examined by ELISA. **(E)** ROC curve of CSF2 expression in osimertinib -sensitive and -resistant LUAD patients. **(F)** Correlation analysis between the expression of CSF2 and osimertinib resistance. The results are presented as the mean ± SD of three technical replicates. **P* < 0.05; ***P* < 0.01; ****P* < 0.001; *****P* < 0.0001, ns = no significance.

Figure S4


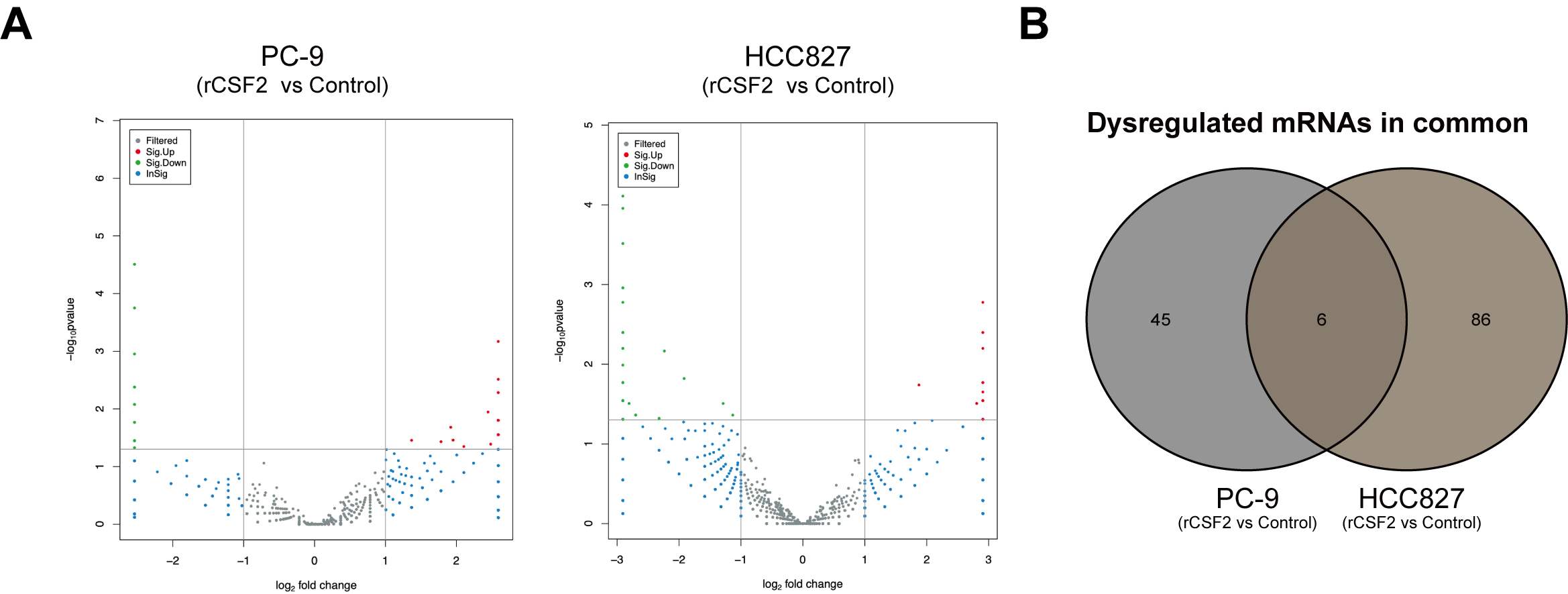


**Fig. S4** The differences in mRNAs after rCSF2 treatment. **(A)** Volcano plot showing the differentially expressed mRNAs (|Log 2 FC | >1, p-value < 0.05) in the RNA sequencing data of PC-9 and HCC827 cells treated with rCSF2 (CSF2-Fc fusion protein) or control (hIgG1 Fc fragments). **(B)** Venn diagram showed the number of differentially expressed mRNAs overlapping between two comparisons.

Figure S5


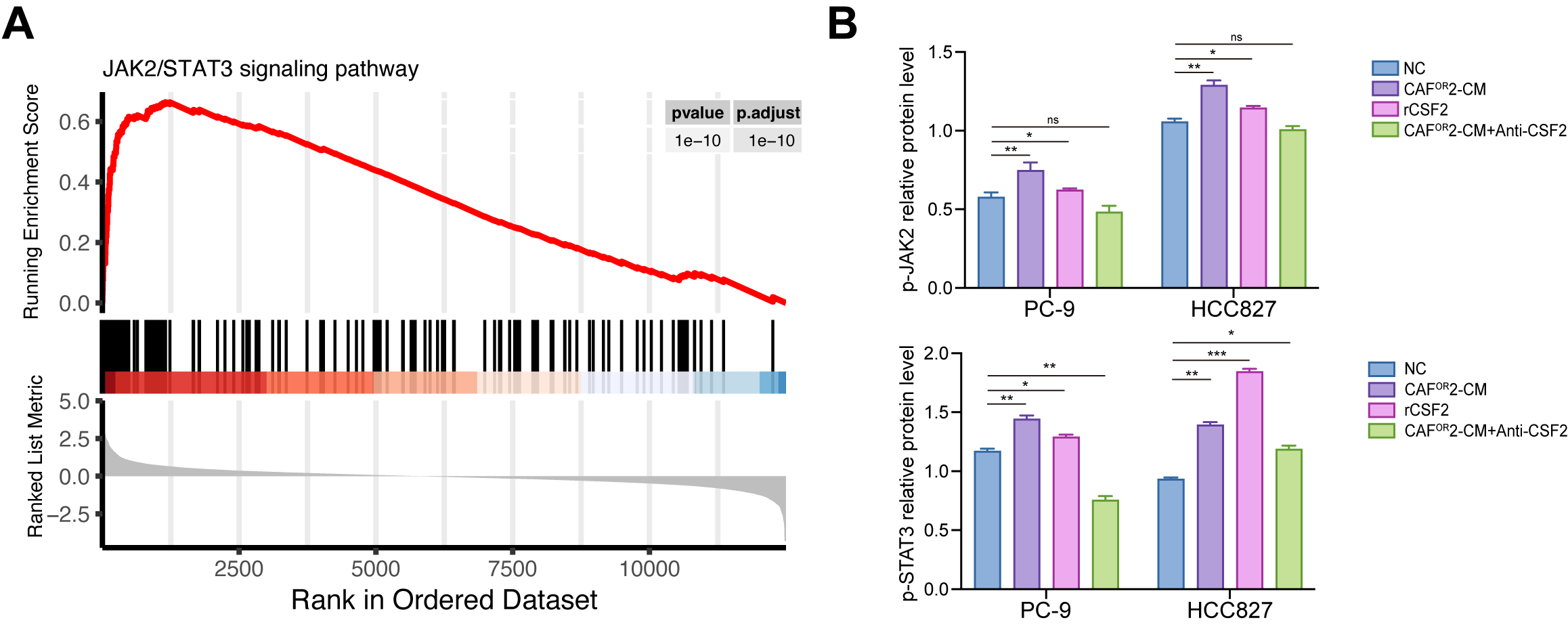


**Fig. S5** CAF^OR^-derived CSF2 activated the JAK2/STAT3 signaling pathway in LUAD cells. **(A)** GSEA analysis of differentially expressed mRNAs after rCSF2 treatment. **(B)** Quantify intensity of p-JAK2 and p-STAT3 relative protein levels in Fig. 5C (p-JAK2, JAK2, p-STAT3, STAT3 protein level was normalized to GAPDH, and p-JAK2, p-STAT3 protein level was normalized to related JAK2 and STAT3). The results are presented as the mean ± SD of three technical replicates. **P* < 0.05; ***P* < 0.01; ****P* < 0.001; *****P* < 0.0001, ns = no significance.

Figure S6


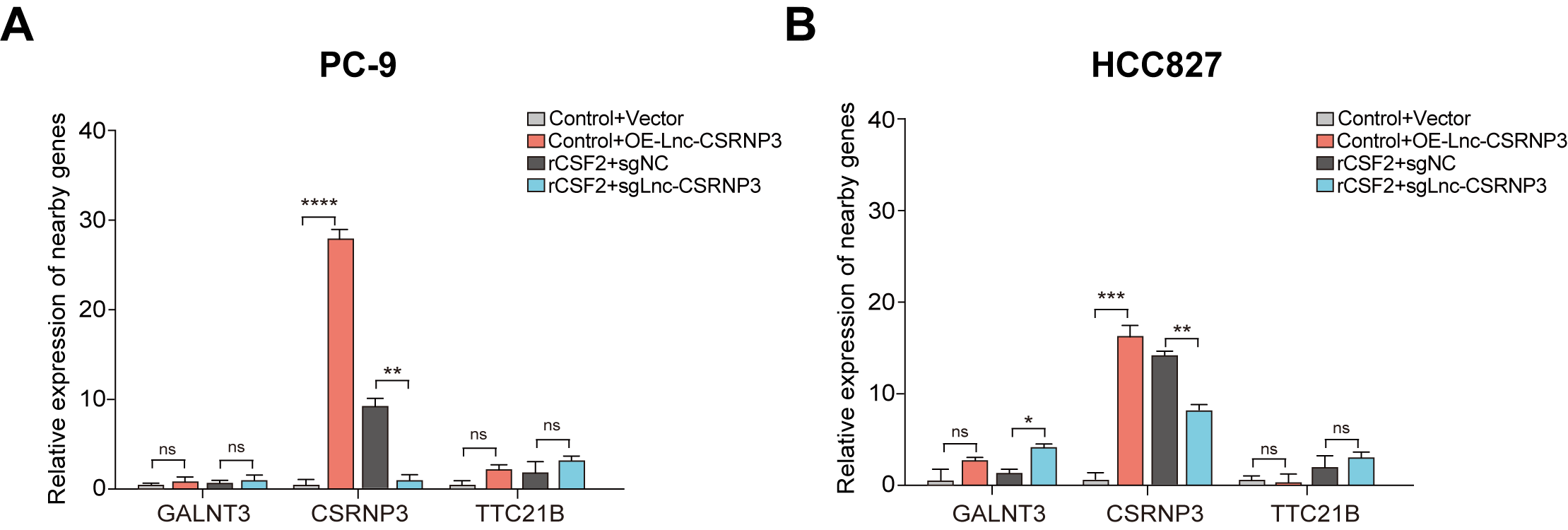


**Fig. S6** CSRNP3 expression was regulated by lnc-CSRNP3. **(A)** Relative expression of nearby genes in PC-9 cells. **(B)** Relative expression of nearby genes in HCC827 cells. The results are presented as the mean ± SD of three technical replicates. **P* < 0.05; ***P* < 0.01; ****P* < 0.001; *****P* < 0.0001, ns = no significance.

Figure S7


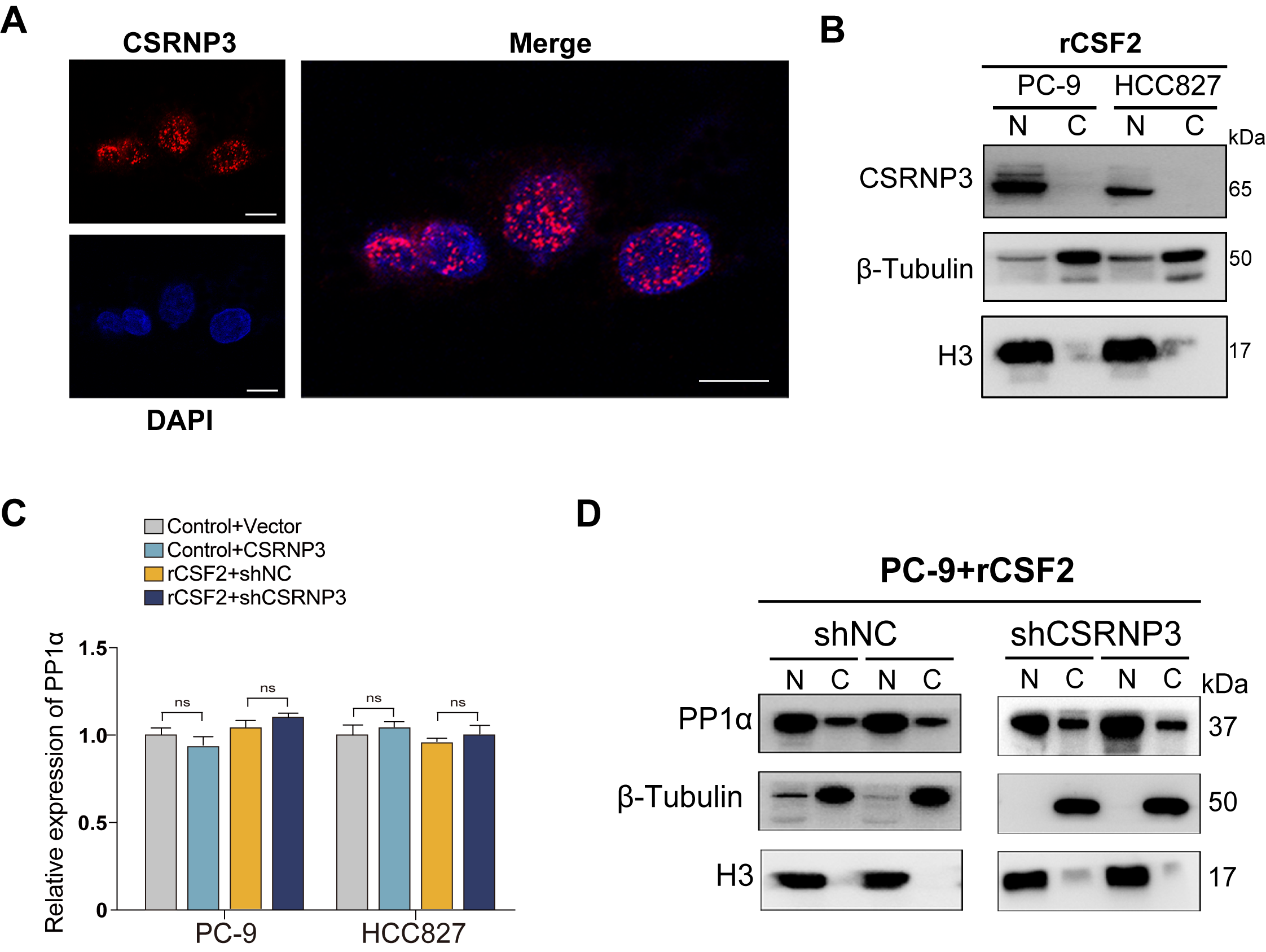


**Fig. S7** CSRNP3 was located in the nuclei and did not influence PP1α expression or cellular distribution. **(A)** Immunofluorescence of CSRNP3 in PC-9/GR showed the nucleus location of CSRNP3. Scale bar = 100 μm. **(B)** Western blotting assay following nuclear/cytoplasmic protein separation showed the subcellular location of CSRNP3. **(C)** CSRNP3 did not influence PP1α expression. **(D)** CSRNP3 did not influence PP1α subcellular distribution. The results are presented as the mean ± SD of three technical replicates. **P* < 0.05; ***P* < 0.01; ****P* < 0.001; *****P* < 0.0001, ns = no significance.

Figure S8


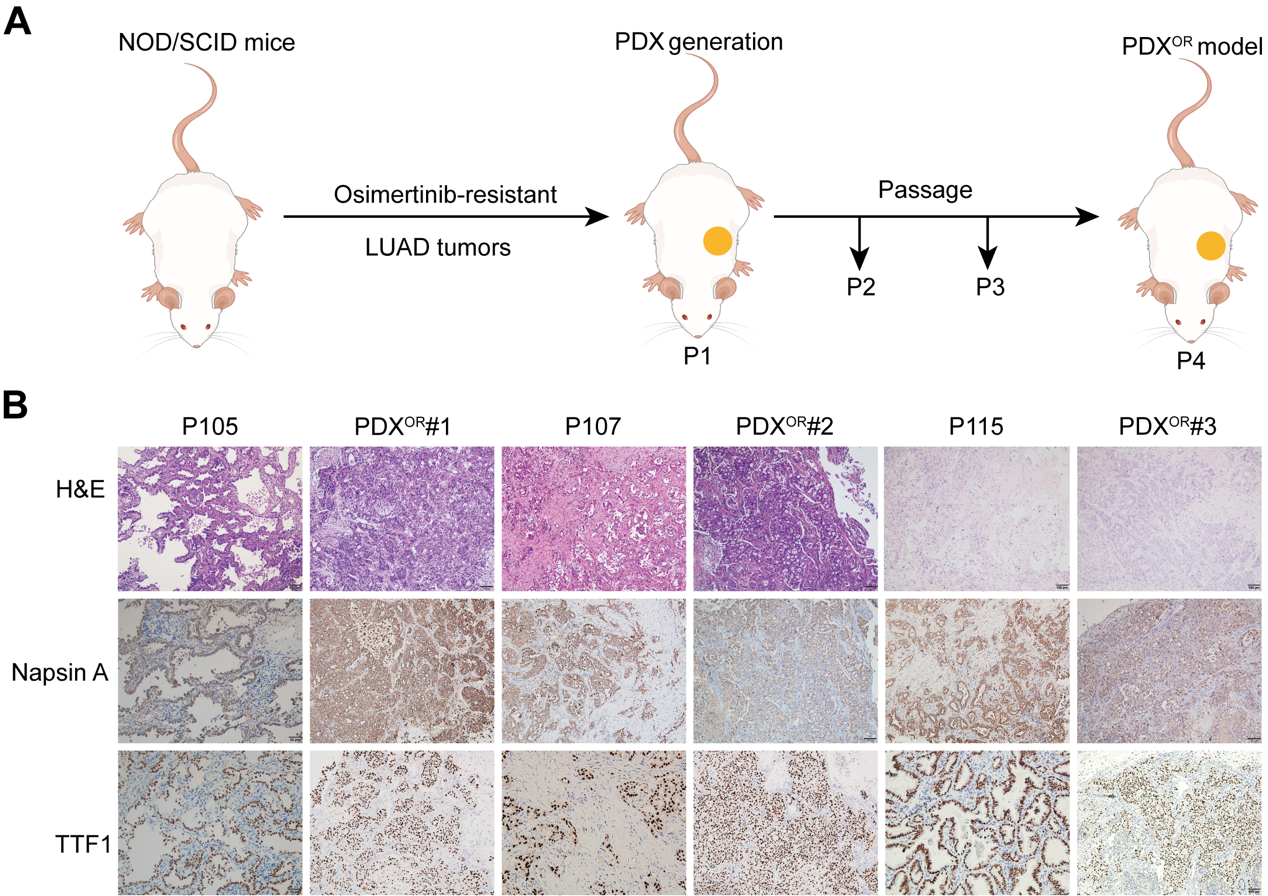


**Fig. S8** Generation and identification of PDX^OR^ model mice. **(A)** Schematic diagram of generating PDX^OR^ model mice. **(B)** The H&E and IHC assays revealed that the PDX models displayed a pathologic phenotype compatible with the tumor tissues from which they were derived. Scale bar = 100 μm.

**Supplementary Tables**

**Table S1. Clinical information of the nine patients enrolled for snRNA-seq.**

|  | P96 | P97 | P105 | P107 | P109 | P112 | P113 | P114 | P115 |
| --- | --- | --- | --- | --- | --- | --- | --- | --- | --- |
| Sex | female | female | male | female | female | female | female | male | male |
| Age | 78 | 58 | 72 | 55 | 68 | 72 | 70 | 55 | 66 |
| Chemotherapy before Osi treatment | No | No | MTA  +NDP | MTA  +NDP | No | No | No | No | MTA  +DDP |
| Clinical AJCC | ADC  (IIA2) | ADC  (IIIA2) | ADC  (IVA) | ADC  (IIIA2) | ADC  (IIA) | ADC  (IB) | ADC  (IB) | ADC  (IA3) | ADC  (IIA) |
| Smoking habit | No | No | Yes | No | No | Yes | No | Yes | No |
| EGFR mutations | Exon  19^del^ | Exon  19^del^ | Exon 21^L858R^ | Exon  19^del^ | Exon 21^L858R^ | Exon  21^L858R^ | Exon  19^del^ | Exon  19^del^ | Exon  19^del^ |
| Drug tolerance | OS | OS | OR | OR | OS | OS | OS | OS | OR |

*MTA, pemetrexed; NDP, nedaplatin; DDP, cisplatin; ADC,* *adenocarcinoma; OS, osimertinib sensitive; OR, osimertinib resistant.*

| Table S2. Clinicopathological factors of plasma samples of LUAD patients (Osimertinib-sensitive/-resistant) and expression of CSF2. | | | | |
| --- | --- | --- | --- | --- |
| Characteristic | N | | | P value |
|  | Total | Sensitive | Resistant |  |
| Age (yr) |  |  |  | 0.7194 |
| ≥ 60 | 16 | 9 | 7 |  |
| < 60 | 14 | 6 | 8 |  |
| Gender |  |  |  | 0.6426 |
| Male | 14 | 6 | 8 |  |
| Female | 16 | 9 | 7 |  |
| Smoking status |  |  |  | 0.2315 |
| Never | 13 | 6 | 7 |  |
| Former | 3 | 1 | 2 |  |
| Current | 10 | 4 | 6 |  |
| Unknown | 4 | 4 | 0 |  |
| Expression of CSF2^*^ |  |  |  | 0.0473 |
| Low expression | 15 | 13 | 2 |  |
| High expression | 15 | 2 | 13 |  |

*The cutoff value of CSF2 was calculated using Youden index (specificity + sensitivity - 1).

**Table S4. Clinicopathological characteristics of LUAD patients in this study (n=30)**

| **Patient ID** | **Age (year)** | **Sex** | **Smoking status** | **CSF2 protein levels in the plasma sample (pg/mL)** | **Osimertinib sensitivity** |
| --- | --- | --- | --- | --- | --- |
| #1 | 68 | Female | Former | 798.883 | R |
| #2 | 58 | Female | Never | 764.752 | R |
| #3 | 54 | Male | Never | 736.221 | R |
| #4 | 65 | Male | Former | 648.226 | R |
| #5 | 57 | Male | Never | 534.900 | R |
| #6 | 50 | Male | Current | 492.769 | R |
| #7 | 61 | Male | Current | 485.036 | R |
| #8 | 67 | Male | Never | 421.840 | R |
| #9 | 75 | Female | Never | 393.042 | R |
| #10 | 78 | Male | Current | 302.381 | R |
| #11 | 70 | Male | Never | 300.781 | R |
| #12 | 57 | Female | Current | 294.915 | R |
| #13 | 53 | Female | Never | 279.983 | R |
| #14 | 55 | Female | Current | 199.454 | R |
| #15 | 59 | Female | Current | 142.924 | R |
| #16 | 62 | Female | Never | 26.132 | S |
| #17 | 58 | Male | Current | 50.664 | S |
| #18 | 56 | Female | Never | 221.853 | S |
| #19 | 73 | Female | Current | 120.792 | S |
| #20 | 71 | Female | Never | 168.256 | S |
| #21 | 66 | Male | Current | 216.253 | S |
| #22 | 62 | Female | Never | 229.852 | S |
| #23 | 68 | Female | Never | 247.451 | S |
| #24 | 76 | Female | Never | 247.985 | S |
| #25 | 53 | Male | Current | 269.850 | S |
| #26 | 54 | Male | Former | 270.916 | S |
| #27 | 57 | Female | Unknown | 273.050 | S |
| #28 | 74 | Female | Unknown | 278.916 | S |
| #29 | 59 | Male | Unknown | 289.049 | S |
| #30 | 65 | Male | Unknown | 291.715 | S |

**Table S5. The primer sequences used in this study.**

| **Target** | **Primer** | **Sequence 5'-3'** |
| --- | --- | --- |
| lnc-ABCA12-5:1 | Forward | AGCTTAATGCGCTTTGCTTT |
|  | Reverse | TGTGGAAGGAACATCCAAGA |
| lnc-ABCA12-8:1 | Forward | TTCCCCCTATTCCAGAGACC |
|  | Reverse | CCTGTGAGAAGCCAGTATTTGA |
| lnc-CSRNP3-6:1 | Forward | CCAGGCACAGTAGCTCATGC |
|  | Reverse | TTGGACAGGGTTTTGTACAGG |
| lnc-FAM150B-3:1 | Forward | TAGGATTGTCTTGCCCATGC |
|  | Reverse | CCTCACCAAGCTACCATTGAC |
| lnc-LRRFIP2-2:2 | Forward | CCTTACCCTTGTTTTTCTTTTCC |
|  | Reverse | CCAACAGGATAGACTTGCCATT |
| CSF2 | Forward | CTGGAGCTGTACAAGCAGGG |
|  | Reverse | CACAGGAAGTTTCCGGGGTT |
| GALNT3 | Forward | GCGTTGGTCAGCCTCTATGTCTG |
|  | Reverse | AACGAGACCTTGAGCAGCATGAAG |
| CSRNP3 | Forward | GCAGCGGAGTCACAGATTCTAGC |
|  | Reverse | CGTCATCCTCCTCCTCCTCTTCC |
| TTC21B | Forward | TGGCACGATTATACCTGGCACAAG |
|  | Reverse | GCAGCTTCGTTATCCTGGTCACTC |
| PP1α | Forward | CCTGCTGGAAGTGCAGGG |
|  | Reverse | GAAGAATGGGCTGGCTCAGA |
| 5S rRNA | Forward | GGCCATACCACCCTGAACGC |
|  | Reverse | CAGCACCCGGTATTCCCAGG |
| 18S rRNA | Forward | AGGCGCGCAAATTACCCAATCC |
|  | Reverse | GCCCTCCAATTGTTCCTCGTTAAG |
| 28S rRNA | Forward | AACGAGATTCCCACTGTCCC |
|  | Reverse | GCCTTAGGACACCTGCGTTA |
| 45S rRNA | Forward | CCTGCTGTTCTCTCGCGCGTCCGAG |
|  | Reverse | AACGCCTGACACGCACGGCACGGAG |
| U6 | Forward | CTCGCTTCGGCAGCACA |
|  | Reverse | AACGCTTCACGAATTTGCGT |
| GAPDH | Forward | TGACGTGGACATCCGCAAAG |
|  | Reverse | CTGGAAGGTGGACAGCGAGG |
|  | | |

**Table S6. Antibodies used in this study.**

| **Antibodies** | **Source** | **Dosage/Dilution** |
| --- | --- | --- |
| Anti-CSF2 (Lenzilumab) | MCE | 10mg/kg |
| p27 | Bioss | 1:1000 |
| Ki-67 | Beyotime | 1:100 |
| Cle-caspase3 | Beyotime | 1:100 |
| CSRNP3 | Bioss | 1:1000 |
| β-Tubulin | Beyotime | 1:1000 |
| CHD9 | Abcam | 1:20 |
| PP1α | GeneTex | 1:50 |
| Flag | Sigma-aldrich | 1:500 |
| p-STAT3 | Abcam | 1:1000 |
| STAT3 | Abcam | 1:1000 |
| p-JAK2 | Abcam | 1:1000 |
| JAK2 | Abcam | 1:1000 |
| p-Rb | Bioss | 1:1000 |
| Rb | Bioss | 1:1000 |
| Puromycin | Sigma-aldrich | 1:1000 |
| β-actin | Abcam | 1:1000 |
| GAPDH | Abcam | 1:1000 |

**Table S7. The sequences of shRNA and gRNA used in this study.**

| **Target** | **Sequence 5'-3'** |
| --- | --- |
| lnc-CSRNP3 gRNA-1 | F: GCAGCTTCTTAATGCTATAT |
|  | R: CTGGAATGATACAGTCACTT |
| lnc-CSRNP3 gRNA-2 | F: CACTTTACTGACTTGAAGAG |
|  | R: TTATCAGTCACATGACCATC |
| sh-CSRNP3-1 | CAGAAGAATGGTACAAATCCAAG |
| sh-CSRNP3-2 | CCCTTAAAGGAACCAATGAGTCC |
| sh-CSRNP3-3 | AGGATGAGATTCAGAATATGAAG |
| si-STAT3 | GAGAUUGACCAGCAGUAUA |
| si-STAT3 | CAACAUGUCAUUUGCUGAA |

**Table S8. The primer sequences of ChIP assay used in this study.**

| Binding sites | Primer sequences |
| --- | --- |
| STAT3 P1 | AGCAAATAAATCCTAGTTTA |
|  | CAAGATTAAAGTGATAACA |
| STAT3 P2 | TCTTTAAAAATAAAATACCA |
|  | TAATTTTTACTTCTAGTTTA |
| STAT3 P3 | TACAGTTTCCCCATTGGCCC |
|  | GGTGATGGGCTTTTATCTCC |
| CHD9 BS1 | F: AATTCAGTAGCGCCTGTAGT |
|  | R: ATATCCTGGATAATTTATGTC |
| CHD9 BS2 | F: ATTCACTCAAAACTTTGAAG |
|  | R: ATATCTAAACGTATATAGAAA |
| CHD9 BS3 | F: CATATACACAGTAAGTCTGTG |
|  | R: AATCAAATTTGAGAAGTAAA |

**Supplementary Materials and Methods**

**Processing of clinical samples**

Whole blood was collected into tubes containing ethylenediaminetetraacetic acid (EDTA) and centrifuged within 4 h to obtain plasma (2,000 g for 10 min at 4 ^o^C). Then, the plasma samples were centrifuged (12,000 g for 10 min at 4 ^o^C) to remove cellular nucleic acids attached to cell debris, and stored at –80 ^o^C until use.

All LUAD tissues were collected from 9 patients who had surgery at the First Affiliated Hospital of Chongqing Medical University. Briefly, tissue samples from the operating room were placed in a 1640 culture medium, transported to the laboratory on ice, and split into four parts for single-nucleus RNA sequencing, protein and RNA extraction, patient-derived tumor xenograft (PDX) model establishment, and immunohistochemistry.

**snRNA-seq analysis**

*Isolation of Nuclei*

The frozen human lung tissue samples were processed according to the “Nuclei extraction and library preparation” protocol described previously 61. Briefly, the tissue was homogenized, and the nuclei were isolated using a density gradient. The nuclei were then sequenced using the 10×Genomics Chromium Next GEM Single Cell 3ʹ Reagent Kits v3.1 (1000268), targeting 10,000 cells per sample and 50,000 reads per cell for each sample.

*Library Preparation and RNA Sequencing*

Library preparation was performed using Chromium™ Single Cell 3'/5' Library Construction Kit (1000020), and cDNA quality was evaluated by fragment analysis (5200 Fragment Analyzer System, Agilent). RNA sequencing was carried out using the Illumina Nova 6000 platform.

*Bioinformatics Analysis*

The sequencing data were processed using the Cell Ranger software pipeline (version 5.0.0). The pipeline was used to first discard reads with low-quality barcodes and UMIs and then, map reads to the human reference assembly. The barcode-gene matrix generated by UMIs was analyzed using the R package Seurat (version 4.0.0). All the sequencing procedures and analyses were performed in OE Biotech Co., Ltd. (Shanghai, China).

*RNA-seq analysis*

Total RNA from LUAD cells treated with CSF2 and IgG was extracted using TRIzol reagent (Invitrogen, USA), and was treated with the Turbo DNA-free Kit (Thermo Fisher Scientific, USA) to degrade the remaining DNA. The RNA was subsequently purified using Ribo-Zero Gold Kit (Illumina, USA) and RNase R (Epicenter, USA). RNA integrity was evaluated using the Agilent 2100 Bioanalyzer (Agilent Technologies, Santa Clara, CA, USA). The samples with RNA Integrity Number (RIN) ≥ 7 were subjected to the subsequent analysis. The libraries were constructed using TruSeq Stranded Total RNA with Ribo-Zero Gold (Illumina, USA) according to the manufacturer’s instructions. Then these libraries were sequenced on the Illumina sequencing platform (HiSeqTM 2500) and 150 bp/125bp paired-end reads were generated. All the sequencing procedures and analyses were performed in OEbiotech (Shanghai, China). The criteria for differential lncRNA expression included fold change ≥2.0 or ≤0.5, P value <0.05, and false discovery rate (FDR) < 0.05.

**Primary human CAFs isolation and culture**

CAFs were isolated from fresh LUAD samples by using a Human Tumor Dissociation Kit (Miltenyi Biotec, Germany). Briefly, the tissues were minced and digested into single-cell suspensions. After ﬁltration with 70 mm cell strainers, the stromal fraction was collected by centrifugation at 250 g for 5 min and incubated with DMEM and 15% FBS. Magnetic-activated cell sorting with anti-FSP (fibroblast-speciﬁc protein) was used to purify the primary human CAFs isolated as indicated above.

**Conditional medium preparation**

The CAFs were refreshed with DMEM and cultured for another 24 hours once they reached 70% confluency. The conditioned medium was collected, followed by centrifugation at 3000 rpm for 10 min. Then the conditioned medium was ﬁltered with a 0.22 μm sterile ﬁlter and stored at −80 °C for further usage. An anti-CSF2 neutralizing antibody and Stattic (MCE, China) were added to each group.

Cytokine antibody array

A cytokine antibody array was performed by using a Human Cytokine Antibody Array C5 (Raybiotech, USA). In brief, the CAF medium was incubated with an array membrane overnight at 4 °C, followed by incubation with detection antibody cocktails for 2 h and streptavidin-HRP for 1 h. Cytokine dots were detected using a chemiluminescence image system (Fujifilm, Japan).

**ELISA**

The ELISA was performed by using CSF2 ELISA kits (ab174448, Abcam, USA) according to the manufacturer’s instructions. Briefly, primary CAFs were cultured in fresh serum-free medium for 24 h. Subsequently, the supernatants were collected and used for ELISA. Each experiment was repeated at least three times.

**CSF2-Fc fusion proteins design, expression, and purification**

The CSF2-Fc fusion proteins were designed, expression, and purification by Leqin Biotechnology Company (Chongqing). Briefly, the DNA sequences of CSF2 and hIgG1 FC fragment were separately amplified by PCR, then the two sequences were spliced to obtain the DNA sequence of CSF2-linker-hIgG1 Fc. The target plasmids were obtained by a seamless cloning of the CSF2-linker-hIgG1 Fc sequence into pcDNA3.1 (-) vector while the control plasmids were cloning of the hIgG1 Fc sequence into pcDNA3.1 (-). The control and target plasmids were transformed into E. coli and sequenced after amplification. The correct plasmids were then transiently transfected into HEK293 cells and cultured in a shaking bed of 37°C, 8% CO2 and 120 rpm for 5 days. The supernatants of cell culture medium, cell lysate supernatants, and cell lysate precipitate were collected, respectively. The target protein was detected by SDS-PAGE and Western blot. The supernatant of HEK293 cells was loaded into the Protein A affinity chromatography column (1 mL) and the column was pre-equilibrated with 10 column volumes of equilibrium buffer (20 mM Tris, 500 mM NaCl, pH 8.0). The flow rate was adjusted to 2 mg/mL. The column was then washed with 5 column volumes of equilibrium buffer and eluted with 0.1 M Glycine (pH 3.0). The desired peaks were collected, dialyzed against PBS (pH 7.4), and preserved for further experiments.

**Fluorescence in situ hybridization (FISH)**

The distribution of lnc-CSRNP3 in tumor cells was detected by FISH Kit (Gene Pharma, Guangzhou, China) according to the manufacturer’s instructions. In brief, the cells were fixed and then hybridized with FITC-labeled lnc-CSRNP3 probes overnight at 37 °C. DAPI was used to stain the nuclei. The fluorescence signals were captured by confocal microscopy (Carl Zeiss AG, Germany). The sequences of the lnc-CSRNP3 probe: 5'-TTTGGACAGGGTTTTGTACAGGTGGTAAAG-3'.

**Dual-luciferase reporter assays**

The indicated regions of the lnc-CSRNP3 and CSRNP3 promoter were directly inserted into the pGL3 luciferase reporter plasmid. A dual-luciferase reporter assay system (E1910, Promega, USA) was used to detect the luciferase activities.

**PLA**

PLA was performed using the DuoLink PLA kit (Sigma-Aldrich, USA) according to the manufacturer’s instructions. Briefly, cells were fixed using 4% paraformaldehyde followed by permeabilization with 0.5% Triton X-100. After treatment with DuoLink blocking buffer, cells were incubated with rabbit anti-CSRNP3 (1:50, Bioss, China) and mouse anti-PP1α (1:100, Abcam, USA) primary antibodies, and oligonucleotide-labeled anti-mouse and anti-rabbit secondary antibodies (PLA probes). Subsequently, cells were treated with the ligation-ligase solution and amplification polymerase solution at 37 °C. To localize PLA signals, cells were co-stained using DAPI, and PLA signals (red puncta) were counted on confocal images.

**Drug susceptibility test**

For cell viability assay, cells were seeded in a 96-well plate at a density of 5×10^3^ cells per well. After 24 h of incubation, cells were treated with various concentrations of osimertinib for 48 h, then 10 µL of CCK-8 solution (Dojindo Molecular Technologies, Japan) was added and incubated for an additional 4 h. The absorbance was measured at 450 nm with a Hitachi F-7000 fluorescence Spectrophotometer (Hitachi High-Technologies Corp, Japan).

For colony formation assay, cells were seeded in a 6-well plate at a density of 1×10^3^ per well, and incubated for 1 week at 37°C. Then, cells were washed twice in PBS, fixed with 4% formaldehyde for 15 min, and stained for 15 min with GIMSA. The colonies (a diameter ≥ 75 µm) were counted in triplicate assays.

For cell cycle assay, cells were seeded in a 6-well plate at a density of 1×10^5^ cells per well, and treated with 500nM osimertinib for 48 h. Then, cells were fixed with chilled 70% ethanol, treated with 100 µL RNase A, and stained with 400 µL Propidium Iodide (PI) at 4°C for 30 min in the dark. Cell cycling was analyzed using a CytoFLEX flow cytometer (Beckman Coulter, CA, USA).

For apoptosis assay, cells were treated with 500 nM osimertinib (TargetMol, Shanghai, China) for 48 h. Cell apoptosis was detected by using an Annexin V-PI staining kit (BMS500FI, Invitrogen, USA). Briefly, the cells were harvested and resuspended in Annexin V binding buffer with FITC-conjugated Annexin V and PI dye for 15 min. Then, the cells were analyzed using a flow cytometer within 1 h. The experiment was repeated three times.

Measurement of cell senescence was conducted by SA-β-gal staining kit (Beyotime, Shanghai, China) following the manufacturer’s instructions. Briefly, cells in a 24-well plate were fixed in 4% formaldehyde for 15 min at room temperature. Then the cells were washed with PBS and incubated overnight at 37°C in SA-β-gal staining solution. After incubation, sections were washed twice in PBS and mounted in glycerol.

**Immunohistochemistry (IHC) and immunofluorescence (IF)**

For the IHC assay, xenografts were fixed in 4% paraformaldehyde, embedded in paraffin, and cut at a thickness of 4 mm. The sections were deparaffinized in xylene and incubated with the primary antibodies (Ki-67, 1:100 dilution, Bioss; P27KIP1, 1:100 dilution, Bioss; Cle-caspase3, 1:100 dilution, Bioss) overnight at 4°C. Three slides per group were observed under a standard light microscope (Olympus BX51, Tokyo, Japan). In each section, 10 areas were randomly selected and scored for the positive cells using an automated image analysis system (Visiomorph, Visiopharm Integrator System, Denmark).

For the IF experiment, cells were incubated with primary antibody (FBL, 1:100 dilution, Bioss; COL1A2, 1:50 dilution, Abcam; IL-6, 1:50 dilution, Abcam;) at 4°C overnight, then incubated with fluorescein-conjugated secondary antibodies and imaged by using a fluorescence microscope (Olympus BX51, Tokyo, Japan).

**Monitoring translation by puromycin incorporation**

Puromycin incorporation was conducted to monitor translation. Briefly, cells were seeded in a 6-well plate, and treated with 5 µg/µL puromycin for 15 min when the cell confluence reached about 70%. Proteins were isolated and western blotting using an anti-puromycin antibody (Sigma-Aldrich, USA).

**Silver-staining of the nucleolar organizing region (NOR) proteins**

Silver-staining of the NOR proteins was performed using an AgNOR Stain Kit (LEGENE, China). Briefly, xenograft sections were deparaffinized and washed twice with PBS, and then AgNOR dyeing liquid was added and incubated at room temperature for 40 min. Finally, slides were dehydrated and mounted, and three slides per group were observed under a standard light microscope (BX53, Olympus). In each section, 10 areas were randomly selected and scored for the positive percentage area of NOR sliver-staining (Visiomorph, Visiopharm Integrator System, Denmark).

**Cell line-derived xenograft (CDX) mice model**

For cell-derived xenograft model establishment, BALB/c-nude mice (females, 4–6 weeks, 16–20 g) were randomized into 2 groups (n=10). One group was subjected to subcutaneous co-injection with the lnc-CSRNP3 knockout PC-9 cell (PC-9 sgLnc-CSRNP3) and CAFOR cell, and another group was co-injection with the sgRNA-only PC-9 cell (PC-9 sgNC) and CAFOR cell (5×10^6^ cells/mouse) in each right flank. When all tumors reached a mean volume of 100 mm^3^, the above two groups were further divided into another two groups (n=5), one group was treated with osimertinib (10 mg/kg) by oral gavage (p.o.) and an anti-CSF2 neutralizing antibody (10 mg/kg) by intraperitoneal injection (i.p.) twice a week for 30 days. Investigators were blinded to mouse treatment conditions at the time of measurement. At the end of the experiments, mice were sacrificed, and the tumors were removed for examination of the parameters of interest.

**Patient-derived xenografts (PDX) mice model**

All animal experiments were approved by the Animal Ethics and Experimental Committee of the Chongqing Medical University and performed according to the National Institutes of Health Guide for the Care and Use of Laboratory Animals. For PDX establishment, nonobese diabetic/severe combined immune deficiency (NOD/SCID) female mice (6-8-week-old) were obtained from Changzhou Cavens Laboratory Animal Co., Ltd (Changzhou, China). Fresh tumor specimens (3-5mm^3^) from nine patients were implanted subcutaneously into the flanks of NOD/SCID mice under anesthesia, and collodion was applied around the skin incision for wound healing. Each patient specimen was implanted into five NOD/SCID mice, then tumor size was measured with calipers once a week, and tumor volume was calculated by using the formula: Volume (mm^3^) = (length×width^2^)/2. When tumor size was > 1cm^3^, the PDX mice were anesthetized with 3% pelltobarbitalum natricum and the tumors were further implanted into another cohort of mice, frozen for molecular and histological analysis.

PDX^OR^ mice were randomized into 4 groups (n=5), and when the tumors reached a mean volume of 100 mm^3^, one group was treated with osimertinib (10 mg/kg) by oral gavage (p.o.) and an anti-CSF2 neutralizing antibody (10 mg/kg) by intraperitoneal injection (i.p.) twice a week for 30 days, with IgG was used as a control. Another group was treated with osimertinib (10 mg/kg) by oral gavage (p.o.) and rRNA synthesis inhibitor CX5461 (50mg/kg) by oral gavage (p.o.) twice a week for 30 days, with saline only (saline) was used as a control. Investigators were blinded to mouse treatment conditions at the time of measurement. At the end of the experiments, mice were sacrificed and the tumors were removed for examination of the parameters of interest.

**Co-IP and LC-MS/MS analysis**

Whole-cell lysate of CSRNP3-Flag overexpressing PC-9 cells was prepared using RIPA P0013 lysis buffer (Beyotime, China) supplemented with protease and phosphatase inhibitors (Roche Diagnostics, Germany). After incubation on ice for 30 min and centrifugation (12,000 g at 4 °C) for 15 min, the supernatant was precleared with Protein A/G-Sepharose at 4 °C for 2 h. CSRNP3-Flag interacting partners were co-immunoprecipitated with anti-Flag antibody covalently coupled to Protein A/G-Sepharose.
